# *In Situ* Investigation of Charge Performance in Anatase TiO_2_ Powder for Methane Conversion by Vis–NIR Spectroscopy

**DOI:** 10.1021/acscatal.1c01998

**Published:** 2021-06-20

**Authors:** Tina Jingyan Miao, Chao Wang, Lunqiao Xiong, Xiyi Li, Jijia Xie, Junwang Tang

**Affiliations:** Department of Chemical Engineering, University College London, Torrington Place, London WC1E 7JE, U.K.

**Keywords:** methane conversion, photocatalysis, photoexcited charge carriers, photoinduced *in situ* spectroscopy, anatase TiO_2_

## Abstract

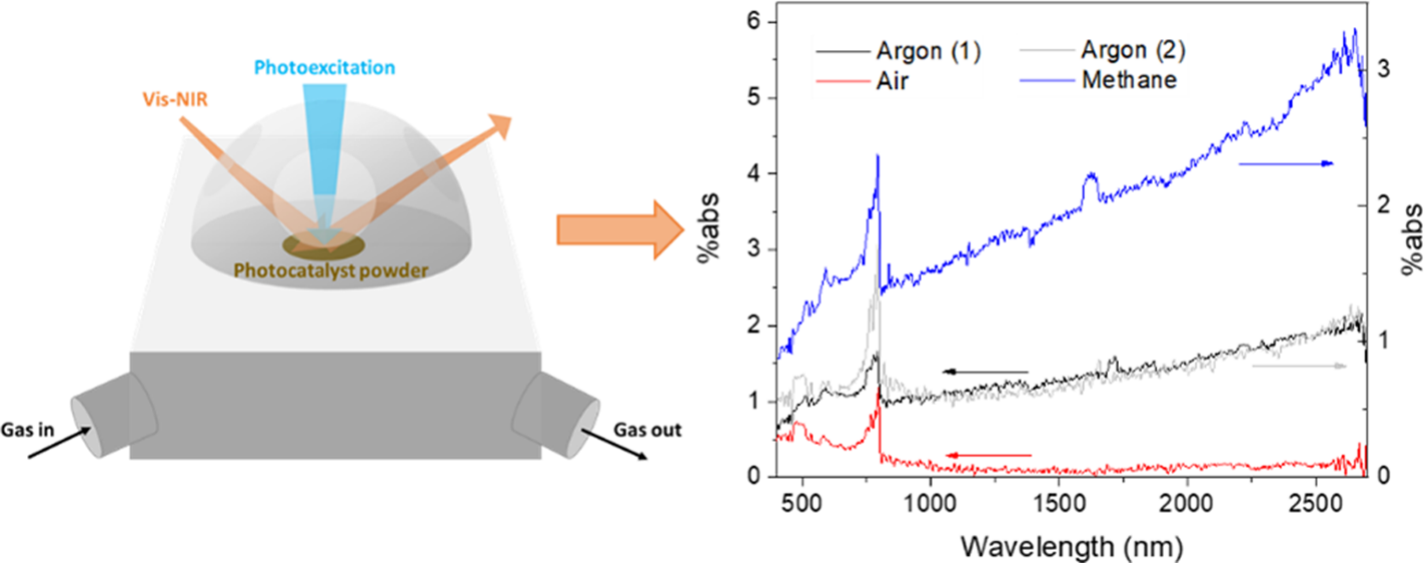

The intrinsic behavior of photogenerated charges and reactions with chemicals are key for a photocatalytic process. To observe these basic steps is of great importance. Here we present a reliable and robust system to monitor these basic steps in powder photocatalysts, and more importantly to elucidate the key issue in photocatalytic methane conversion over the benchmark catalyst TiO_2_. Under constant excitation, the absorption signal across the NIR region was demonstrated to be dominated by photoexcited electrons, the absorption of photoexcited holes increases toward shorter wavelengths in the visible region, and the overall shapes of the photoinduced absorption spectra obtained using the system demonstrated in the present work are consistent with widely accepted transient absorption results. Next, *in situ* measurements provide direct experimental evidence that the initial step of methane activation over TiO_2_ involves oxidation by photoexcited holes. It is calculated that 90 ± 6% of photoexcited electrons are scavenged by O_2_ (in dry air), 61 ± 9% of photoexcited holes are scavenged by methane (10% in argon), and a similar amount of photoexcited electrons can be scavenged by O_2_ even when the O_2_ concentration is reduced by a factor of 10. The present results suggest that O_2_ is much more easily activated in comparison to methane over anatase TiO_2_, which rationalizes the much higher methane/O_2_ ratio frequently used in practice in comparison to that required stoichiometrically for photocatalytic production of value-added chemicals via methane oxidation with oxygen. In addition, methanol (a preferable product of methane oxidation) is much more readily oxidized than methane over anatase TiO_2_.

## Introduction and Background

Photocatalysis is a promising sustainable and green technology with a wide range of applications, including solar fuel production,^1−5^ organics conversion,^3,4,6−8^ and air and water purification.^9,10^ However, the current efficiency in these photochemical processes is very moderate. It is widely accepted that the behavior of photoexcited charge carriers in photocatalysts directly influences this efficiency. As such, the monitoring of charge carriers is the key to provide a fundamental understanding of the performance of different photocatalytic materials and therefore to aid in the rational design of efficient photocatalysts.

Among these photocatalytic processes, the photocatalytic conversion of methane, the main component of natural gas and shale gas, to high-value chemicals has recently attracted much attention in the literature. As photocatalysis provides a rational partial oxidation process to achieve the activation of the highly symmetrical and stable methane molecule under near-ambient conditions, it avoids the increase in the reverse reaction resulting from the conventional thermal–catalytic strategies operated at high temperatures. Therefore, the photocatalytic processes were able to drive the reaction more efficiently with much lower energy consumptions and CO_2_ emissions in comparison to thermal–catalytic pathways. A variety of photocatalysts in the form of powders have been reported to facilitate methane conversion into a range of value-added products such as C2-hydrocarbons, alcohols, and aromatic molecules.^7,8,11−15^ However, fundamental studies, especially from the viewpoint of charge dynamics, are very limited.

Transient absorption spectroscopy (TAS) is a powerful technique for characterizing the behavior of photoexcited charges and has been extensively applied to elucidate charge carrier dynamics in many photocatalysts. While transmission-mode TAS is fairly commonplace to monitor charge carriers in photocatalyst suspensions and films, the application of reflectance-mode TAS is still very limited due to challenges such as difficulty in obtaining good-quality data from light diffusely reflected from solid powders. However, the majority of photocatalysts have been developed as powders and it is very challenging to make good-quality films using these powdered samples. In particular, all of the photocatalysts developed so far for methane conversion are in the form of powders. Furthermore, charge dynamics in the film of photocatalysts could be different from that in the powders due to varying crystal boundary conditions; therefore, an effective and reliable method for characterizing photoexcited charge carriers in powder samples is highly sought after and would be very useful for directing photocatalyst design.

There have been numerous suggestions about the activation of the highly symmetrical and stable molecule methane. First, it was suggested that CH_4_ was able to be directly oxidized by photoexcited holes (e.g., in the form of O^–^ species) in the valence band (VB) of TiO_2_ or TiO_2_-based materials to form the CH_3_^•^ radical and OH^–^^16,17^ or H^+^.^8,17−22^ Also, Yoshida et al. extrapolated knowledge from thermal catalysis and suggested that O^–^ (photoexcited hole) directly extracts a H^•^ radical from CH_4_.^23^ In the case of OH^–^, the electron density mainly resides on the oxygen and therefore can be regarded as a “filled” hole in/near the VB. In contradiction to the above literature, Suzuki et al. reported a computational study which found that direct charge transfer between anatase TiO_2_ and adsorbed methane does not take place.^24^ Consistent with the computational study by Suzuki et al.,^24^ for methane conversion in the presence of H_2_O it has been proposed that H_2_O is first oxidized to the OH^•^ radical by photoexcited holes and then OH^•^ reacts with CH_4_ (i.e., there is no direct charge transfer between methane and photoexcited TiO_2_).^14,25^ In addition, Yoshida et al. observed no H_2_ production when pure TiO_2_ was irradiated under a CH_4_ atmosphere,^26^ which could support the computational finding by Suzuki et al.^24^ However, Kaliaguine et al. observed the formation of ethane and CO_2_ upon UV irradiation of TiO_2_ under pure methane,^16^ which suggests that (i) there is direct interaction between photoexcited charges and CH_4_ and (ii) lattice oxygen is consumed during the photocatalytic process. Following from this, the absence of of H_2_ formation over pure TiO_2_ reported by Yoshida et al.^26^ was likely due to the lack of H^+^ reduction by the TiO_2_ conduction band (CB) electrons. In addition, the computational study by Suzuki et al.^24^ only investigated the anatase TiO_2_(001) surface; therefore, charge transfer between other surfaces and methane may still take place theoretically.

Between the two extremes discussed above, Tahir et al. suggested that both direct oxidation of CH_4_ by valence band holes and indirect oxidation by OH^•^ radicals can take place during TiO_2_-based photocatalysis.^17^ However, Tahir et al. were studying dry reforming of methane (DRM) and therefore the OH^•^ species they proposed likely involved a lattice oxygen, rather than originating from H_2_O. In addition to the methane activation mechanisms discussed thus far, Lang et al. recently reported that the first step occurring during nonoxidative coupling of methane over Au-loaded TiO_2_ was CH_4_ reduction by photoexcited electrons in Au to form the CH_3_^–^ anion and atomic H,^27^ though it is not clear whether this mechanism might also be applicable for pure anatase TiO_2_.

All of the afore mentioned literature proposed the initial interaction between TiO_2_ and methane on the basis of extrapolation of observations made on other photocatalysts,^16^ extrapolation of observations made for thermal catalysis,^23^ deductions based on observed intermediates/products,^14,19−22,25^ computational results,^24^ other literature,^17^ or theoretical scientific reasoning/conjecture.^8,18^ Overall, there has been a surprising lack of direct experimental evidence for the way in which methane interacts with photoexcited charge carriers even in the benchmark photocatalyst TiO_2_. The present study therefore aims to provide fundamental insight into this significant reaction.

As noted above, all photocatalysts used for methane oxidation are in the form of powders and an *in situ* approach to monitor charge characteristics in these powder samples and their reactions with reactants is significant and urgently needed. Steady-state UV–vis–NIR diffuse-reflectance spectroscopy was thus employed to observe the charge behaviors on the powdered benchmark photocatalyst TiO_2_. This technique is analogous to the time-zero measurement in TAS,^28,29^ or photoinduced absorption spectroscopy (PIAS).^30^ These can be broadly labeled as “pump-probe” techniques, in that a “pump” light is used to photoexcite the sample and a “probe” light is used to measure the transmittance/reflectance of the sample in its ground and excited states. Through an analysis of the probe light intensity changes associated with the ground state and excited samples, photoinduced absorption can be calculated, which provides information on the photoexcited characteristics of the sample. The cause of photoinduced absorption is due to electrons and holes introduced in the conduction and valence bands, respectively, upon photoexcitation. The fingerprints of the photoelectron and holes can then be identified by using an efficient charge scavenger. Our recent review provides further background on optical pump–probe techniques and signal interpretation in the context of heterogeneous photocatalysts.^28^

Usually pump–probe techniques are used to obtain fast temporal information (e.g., rate of recombination), and the setup demonstrated in the present work is steady state, in that the sample is measured both in the dark and under continuous excitation (rather than under pulsed excitation, which allows the rate of sample relaxation to be measured). Therefore, the setup used in the present work can be described as *in situ* steady-state photoinduced-absorption diffuse-reflectance spectroscopy. All experiments were performed under controlled atmospheres, including argon (inert reference), dry air (standard electron scavenger), and methanol vapor (standard hole scavenger and a potentially preferable product of methane oxidation). These measurements under standard scavenger conditions demonstrate the capability of the UV–vis–NIR spectrometer to measure the *in situ* absorption spectra of photoexcited charges in powder samples. More importantly, the current design can quantify the ratio of both electrons and holes reacting with methane and oxygen gas, providing a strong indicator for photocatalyst modification for methane conversion.

## Experimental Section

### Design of the *In Situ* Methane Conversion System

A Cary 5000 UV–vis–NIR spectrometer fitted with a Praying Mantis accessory was set up for the measurements. A Harrick Reactor was used to control the sample environment and monitor *in situ* charge behaviors. All three windows of the reaction chamber were 2 mm thick crystal auartz (Crystran, QPZ15-2), transparent for UV, visible, and near-IR (NIR) irradiation. Two of the windows are for the transmission of measurement light, and the third window is for the excitation light. A 300 W Xe lamp (Newport, Model 67005) was used as the excitation light source. The Xe lamp illumination was filtered with a 325–385 nm band-pass filter (Thorlabs, FGUV UG1) and a 365 nm band-pass filter (Comar) and then focused with a lens onto the sample surface. The illumination intensity of the focused 365 nm output was tuned to be ca. 1 mW/cm^2^ around the sample position (the way in which this estimate was made is outlined in section I in the Supporting Information), close to the intensity of 1 sun at this wavelength. To prevent the 365 nm excitation light scattered by the sample from saturating detectors in the Cary 5000 instrument, a 395 nm long-pass filter was taped before the sample beam detector (between the sample and the detector). It is noted here that the two aforementioned band-pass UV filters were employed to ensure clean 365 nm irradiation.

All gases used originate from gas cylinders, delivered to the reactor via copper pipelines. The gas flow rate of each gas was independently controlled using individual Bronkhorst mass flow controllers. The reactor gas inlet and outlet were both fitted with a Swagelok ball valve to allow gases to be sealed in the reactor.

### Measurement Details

Prior to each measurement, the TiO_2_ powder sample (Sigma anatase PC50 with a surface area of 40–50 m^2^/g)^31−33^ was treated at 400 °C for 30 min to remove organic contaminants possibly adsorbed onto the sample surface. This treatment procedure is not expected to induce any phase transitions,^34^ which is supported by XRD patterns of the samples before and after treatment (Figure S1), indicating that the sample remains anatase TiO_2_. The treated powder was transferred into the reaction chamber sample holder and gently patted flat with a spatula.

For each measurement in the presence of Xe lamp illumination, the Xe lamp was switched on for at least 10 min prior to starting the measurement to enable a relatively stable irradiation. To minimize temperature fluctuations, rather than switching the lamp off to perform dark measurements, a sheet of matte black aluminum foil was used to block the Xe lamp output (for a minimum of 5 min) prior to starting a dark measurement. For all measurements, a sheet of blackout fabric (Thorlabs, BK5) was draped over the system to prevent the ceiling lights from possibly interfering with the measurements.

O_2_ is a well-known electron scavenger,^35−39^ delivered in the form of (dry) air in the present experiment. Methanol is a frequently used hole scavenger^35,37,40^ and a preferable product of methane oxidation, delivered as vapor into the reaction chamber by flowing 150 mL/min argon through a metal vessel containing a small amount of liquid methanol. For measurements under a methane atmosphere, 10% methane in argon was used. To simulate conditions required for methane oxidation with oxygen, measurements were also performed under a methane/air mixture. with the flow rates of 10% methane (in argon) and air (ca. 20% O_2_) respectively set to 120 and 15 mL/min, thus resulting in a gas mixture composed of CH_4_/O_2_ in a ratio of approximately 4/1.

For all measurements presented, the pretreated TiO_2_ powder was kept in the dark and purged with 150 mL/min of gas (135 mL/min for the 4/1 CH_4_/O_2_ mixture) for a minimum of 30 min to obtain an adsorption/desorption balance prior to starting the first measurement under that gas environment, and the controlled atmosphere was sealed in the reactor at the end of the purge period by closing the two valves for gas into and out of the reactor. For all experiments, measurements were first performed under an argon atmosphere, and then a reactive gas (mixture) was introduced. The catalyst sample was not reused after the reactive gas measurement in order to avoid the influence of the reaction intermediates adsorbed on the surface of the catalyst on subsequent observation, and another reliable sample was used for the next pair of measurements (argon followed by another reactive gas). Further measurement details are described where appropriate in the Results and Discussion and in the Supporting Information.

## Results and Discussion

### Calculation of Photoinduced Absorption (PIA)

In all conducted measurements, raw %*R* spectra (no baseline) were acquired to allow maximum flexibility in later data processing. There are multiple different ways to process diffuse reflectance data. The Kubelka–Munk (*F*(*r*)) and log(1/*r*) transformations are the two most commonly used functions to convert %*R* values into units reportedly proportional to the concentration of absorbing species. The log(1/*r*) transformation results in “normal” absorbance units, while the *F*(*r*) transformation results in Kubelka–Munk (KM) units.

Steady-state spectrometers are frequently used to measure ground-state samples, for which the KM transformation is often used to process diffuse reflectance data. The KM theory assumes an isotropic homogeneous distribution of absorbers/scattering centers in the sample.^41^ This is evident from the assumption in the original derivation of the KM theory that the absorption and scattering coefficients are constant throughout the entire sample.^42^ However, in the case of a photoexcited powder, this assumption is not generally valid. This is because the excitation light is usually at a wavelength with energy higher than the material’s band gap while the measurement wavelength is usually at sub-band-gap wavelengths. As such, the intensity of the excitation light attenuates more quickly than the intensity of the measurement light; thus, the concentration of absorbers (photoexcited charge carriers) within the sample is inhomogeneous and nonisotropic within distances traveled by the measurement light.

There is limited literature on the steady-state spectroscopic characterization of photoexcited TiO_2_ powder using the diffuse reflectance technique. Liu et al. used the diffuse reflectance infrared Fourier-transform spectroscopy (DRIFTS) technique to study photocatalytic CO_2_ reduction with H_2_O on TiO_2_.^43^ Similar to the present experiment, a Praying Mantis accessory with two windows for the measurement light and a third window for the excitation light was used. However, Liu et al. focused on chemical changes/intermediates characterized by changes to specific IR absorption bands under continuous photoexcitation and reported their spectra in normal absorbance units,^43^ whereas the present experiment focuses on baseline changes associated with absorption by photoexcited charge carriers. DRIFTS has also been used by others to study photoexcited TiO_2_, with the spectra reported in normal absorbance units^44^ or in KM units.^45^ In contrast to these literature reports, the present experiment uses visible to near-IR (NIR) light to measure charge performance in the sample other than reaction intermediates, and focuses on differential reflectance/absorbance for measurements made with and without photoexcitation to characterize photoexcited charges.

When photoexcited charge carriers are the species of interest, it is informative to translate %*R* data into a quantity that is directly proportional to the concentration of excited charge carriers. A commonly used equation to quantify absorption by excited states is^46−54^
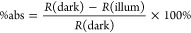
1where %abs is percentage absorption, *R*(dark) is the reflectance of the ground-state sample in the dark, and *R*(illum) is the reflectance of the photoexcited sample. Equation 1 has been extensively used to analyze TA data acquired using diffuse reflectance^46−54^ and is also applicable for analyzing data acquired in the present experiment, because the present experiment is analogous to the time-zero measurement in TAS. However, it is noted that the present system is more similar to PIAS than to TAS, but as far as the authors are aware of there have thus far been no reports of diffuse-reflectance PIAS in literature.

Processing %*R* data using eq 1 is crucial because it has been reported to yield values that are directly proportional to charge carrier concentrations for sufficiently small values of %abs (from here on, this photoinduced absorption signal will be abbreviated as PIA), with <10% being a safe general ballpark estimate.^47,49−51,54,55^ As such, in the present study eq 1 is the primary method used to process %*R* data.

For each condition measured, a total of 4–5 absorption spectra (i.e., 8–10 pairs of raw reflectance spectra) were obtained for each TiO_2_ sample under each condition. To mitigate the effects of instrumental baseline drift, *R*(dark) and *R*(illum) were measured in pairs. Further details can be found in section III in the Supporting Information. Raw reflectance data used to calculate photoinduced absorption of TiO_2_ under air, methanol, methane, and 4/1 CH_4_/O_2_ are respectively shown in Figures S2–S5. It may be observed that the reflectance of photoexcited TiO_2_ increases approximately back to its initial value after 5 min in the dark in the presence of argon, air, methane, and 4/1 CH_4_/O_2_—as shown by data presented in Figures S2, S4, and S5. However, in the presence of methanol, the sample reflectance is still significantly reduced even after 5 min in the dark. This is evident from the data shown in Figure S3. Furthermore, at the end of measurement under all conditions other than methanol, the sample appears unchanged, but in the presence of methanol the sample turns light blue, which is characteristic of Ti^3+^,^56^ indicating that a significant proportion of photoexcited electrons with greater than minutes lifetimes remain in the sample when the efficient hole scavenger methanol is added. As such, although repeats performed on the same sample can be averaged over for measurements made under argon, air, methane, and 4/1 CH_4_/O_2_, such averaging is not appropriate for measurements made in the presence of methanol. Following from this, the results presented in subsequent sections for TiO_2_ are averaged data for measurements made under all conditions other than methanol, while only data from the first repeat is presented for measurements made under methanol.

### Control Experiments: BaSO_4_ Measurements

**Figure 1:**
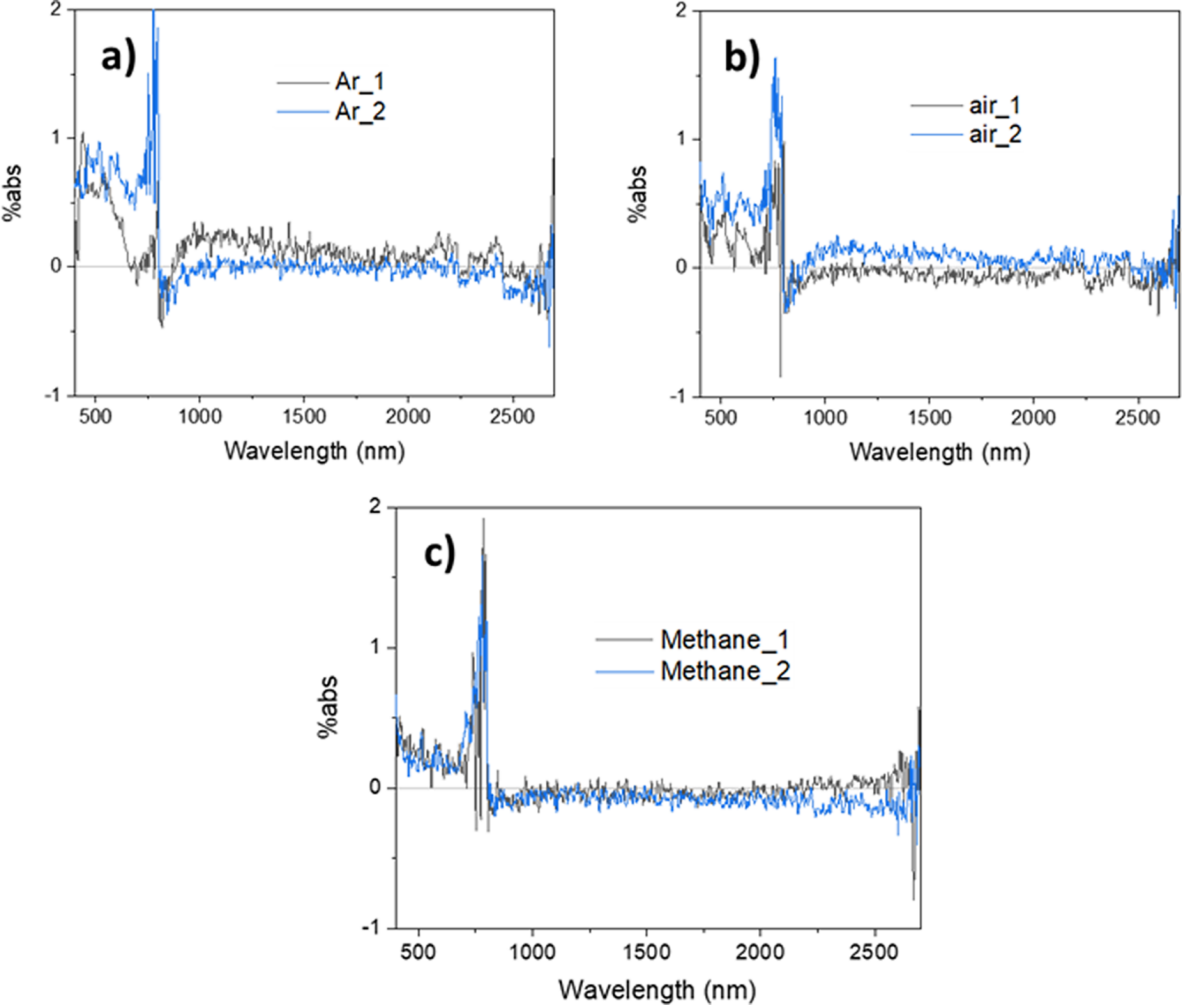
Absorption (PIA) induced by 365 nm external illumination for BaSO_4_ powder under (a) 100% argon, (b) dry air, and (c) 10% methane in argon atmospheres. Legends are in the form “C_*x*”, where C represent the gaseous environment, and *x* represents the repeat number. The spectrometer can measure up to 3300 nm, but in the present setup the noise is very large at long wavelengths; therefore, only reliable data up to 2700 nm are shown. The discontinuity at 800 nm is due to the detector and grating changeover at this wavelength.

Control experiments were performed using commercial BaSO_4_ powder as the reference sample to validate the current system. Further measurement details can be found in section IV in the Supporting Information. The absorptions induced by the 365 nm external illumination for BaSO_4_ under different gaseous environments are shown in Figure 1. A 365 nm illumination is not expected to generate any excited charge carriers in BaSO_4_ due to its extremely large band gap corresponding to ∼200–300 nm;^57,58^ therefore, the ideal photoinduced absorption spectrum should be a straight line at 0% under all presently introduced gases. The spectra in Figure 1 slightly deviate from the ideal 0% line due to noise and small instrumental drifts/errors. However, overall it may be observed that there is no consistent absorption artifact. Deviations from the expected 0% absorption are relatively pronounced in the visible region but are very small (less than 0.5%) in the NIR region, and there is a discontinuity at 800 nm due to the detector and grating changeover at this wavelength. From Figure 1, it may be concluded that PIA features with magnitudes of less than 1% in the visible region can be attributed to instrumental artifacts, while significant artifacts are not expected in the NIR region. Also, there is no significant difference between spectra acquired under different gaseous environments (Figure 1), and the absorption spectra are more or less reproducible; therefore, the current system behaves as expected and can be reliably used to characterize photoexcited charges in powders.

### Fingerprints of Charge Carriers in TiO_2_

**Figure 2:**
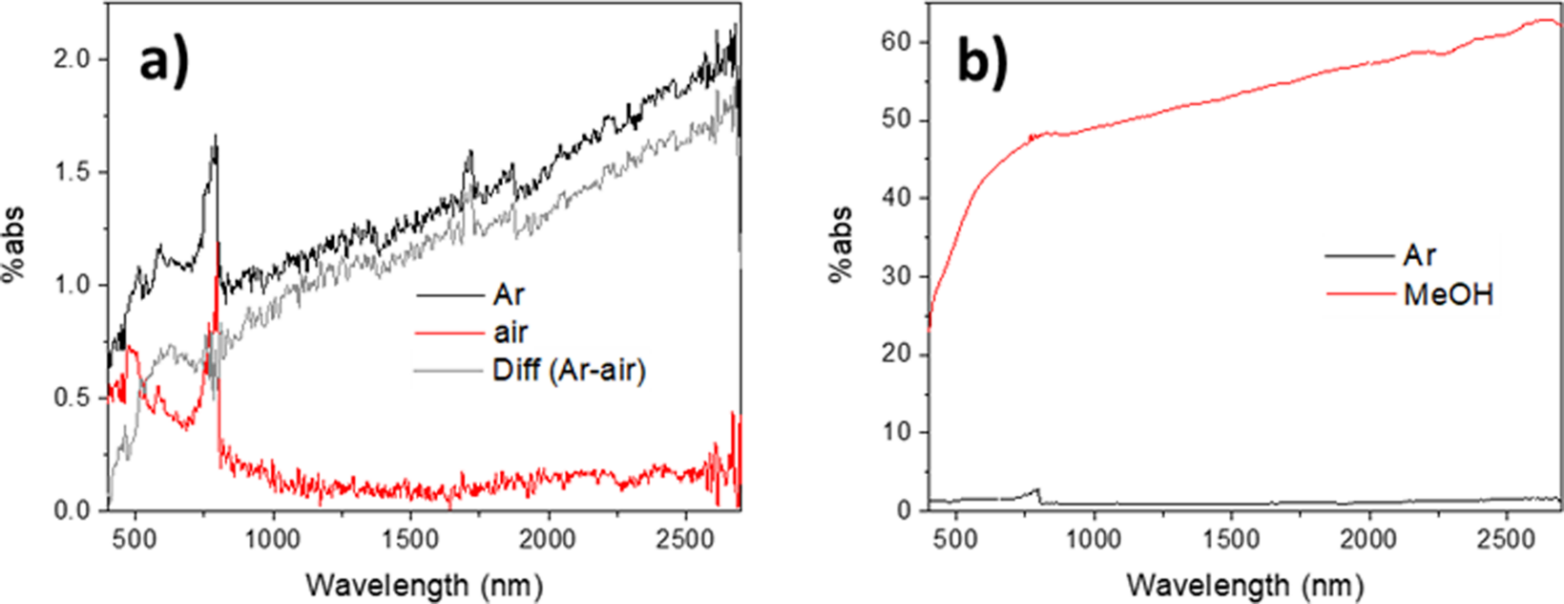
Photoinduced absorption spectra (PIA) of anatase TiO_2_ powder under 365 nm excitation in the presence of (a) air and (b) methanol vapor in argon. A reference spectrum acquired under 100% argon is also shown in each panel for comparison. In (a), the difference spectrum between the two spectra collected under different atmospheres is also shown in gray. The discontinuity at 800 nm is an artifact occurring at the detector and grating changeover wavelength. The average excitation intensity is estimated to be around 1 mW/cm^2^. Apart from the MeOH trace in (b), all other traces are the average of several repeats on the same sample (three repeats for the data shown in (a) and five repeats for the argon trace in (b)). Data for individual repeats can be found in Figures S6 and S7 in section V in the Supporting Information for traces in (a) and (b), respectively.

Taking our previous observation of charge carriers in TiO_2_ by TAS as a reference,^40^ here we first observed the charge characteristic in TiO_2_ under argon and then in the presence of different scavengers to abstract the fingerprints of charge carriers in TiO_2_. When Figure 2a is compared to the control data in Figure 1, it may be observed that a significant absorption signal that increases toward longer wavelengths is induced by 365 nm excitation of TiO_2_ under argon. Also, Figure 2a shows that, when argon is replaced by (dry) air, the photoinduced absorption at wavelengths >1000 nm decreases by about 1 order of magnitude. As such, signals in this region can be assigned to photoexcited electrons that can relatively easily react with O_2_. The magnitude of signal quenching by O_2_ decreases toward shorter wavelengths (<1000 nm), for which there are two possible reasons: (1) the signal contribution from photoexcited holes increases while contributions from photoexcited electrons decreases toward shorter wavelengths or (2) signals at shorter wavelengths are due to deeper trapped electrons that have smaller reductive potentials and therefore are less capable of reducing O_2_. The first reason is consistent with literature reports that absorption due to photoexcited holes in anatase TiO_2_ exhibit a broad peak around 400–500 nm,^37,38,40,59^ while absorption due to photoexcited electrons increase toward longer wavelengths all the way from the visible to the IR region.^37,40^ The second reason is consistent with the finding that conduction band (CB) electrons are quenched by O_2_ more efficiently in comparison to trapped electrons.^60^

When methanol vapor is introduced as a hole scavenger, the photoinduced absorption increases by ∼50 times across the entire 400–2700 nm region, as shown in Figure 2b. This indicates that there are significant signal contributions from photoexcited electrons in the entire 400–2700 nm region. This appears to be different from signal contributions observed by TAS, in which the absorption due to photoexcited holes were >3 times greater than the absorption due to photoexcited electrons at ca. 500 nm in 20 μs after photoexcitation.^40^ If the photoinduced signal in the visible region is mainly dominated by holes and the average excited electron/hole concentrations are similar under argon and under methanol, then upon removal of holes by methanol the overall signal magnitude should decrease in the visible region. Although the opposite is presently observed, this cannot confirm that the photoinduced signal in the visible region is dominated by electrons, because it could be that the population of photoexcited electrons increased so much that it counteracts the decrease in signal associated with hole removal. On the basis of TAS spectra, it is likely the case that the photoinduced signal in the visible region is dominated by holes. Nonetheless, the possibility that the photoinduced signal in the visible region is dominated by (deep-trapped) electrons cannot be dismissed.

In the present experiment, photoinduced absorption is characterized using continuous excitation, whereas in TAS pulsed excitation is used and the reported spectra are usually acquired after some short time delay. As such, if it is the case that the photoinduced signal in the visible region is dominated by (deep-trapped) electrons (rather than holes), the discrepancy between the present findings and literature TAS results could be because electrons that absorb in the visible region exhibit very fast decay, so their signal is only significant at time zero (in TAS). In addition, the dramatically enhanced signal of photoelectrons under methanol can be attributed to the accumulation of excited trapped electrons due to the use of continuous excitation in the current setup, different from the pulsed excitation used in TAS.

In addition, it may be observed from Figure 2b that the signal amplitude gradually varies with wavelength in the region >800 nm, but in the region <800 nm the signal amplitude rapidly decreases with decreasing wavelength. This could be due to two possible reasons: (1) the extinction coefficient due to photoexcited electrons rapidly decreases with decreasing wavelength in the visible region and/or (2) electrons mainly responsible for signals in the <800 nm region are intrinsically different from those responsible for signals in the >800 nm region and electrons that absorb in the visible region undergo faster recombination, resulting in a lower average concentration of these electrons. The latter is consistent with earlier conjectures that the signals at shorter wavelengths are due to more deeply trapped electrons and that electrons primarily responsible for signals in the visible region exhibit very fast decay kinetics. This is also consistent with the suggestion that shallow traps facilitate charge migration and therefore enhance photocatalytic activity, while deep traps increase recombination.^61^

Normalizing the spectra in Figure 2b reveals that the spectral shapes measured under argon and methanol vapor significantly differ from one another, as shown in Figure S7Ba,Bb. In the visible region, there is a slight peak between 500 and 650 nm for the spectra measured under argon, while the signal amplitude rapidly decreases with decreasing wavelength for the spectra measured under methanol. In the NIR region, the slope/curvature of the spectra measured under methanol slightly differs from the slope/curvature of the spectra measured under argon. Under argon, photoexcited electrons and holes are expected to contribute equally to the photoinduced signal, while in the presence of methanol, only signals from electrons are significant. As such, the differing spectral shapes in the visible region observed under argon and methanol are strong evidence that the photoinduced signal observed under air (more prominent absorption toward shorter wavelengths in Figure 2a) is primarily due to holes and that the photoinduced signal in the visible region under argon has significant contributions from holes. Nonetheless, there is still some (likely very little) possibility that the photoinduced signal in the visible region is dominated by (deep-trapped) electrons—if the signals in the NIR and visible regions are respectively attributed to shallow/free electrons and deep-trapped electrons, the difference between the normalized spectra in the visible region could suggest that the populations of shallow/free electrons are enhanced more than the populations of deep-trapped electrons. This could be understood as there being a limited small number of deep trap states compared to shallow/free states. Different from the comparative spectral shapes under argon and methanol in the visible region discussed thus far, the spectral shapes in the NIR region are similar with only small differences. Also, following from earlier discussions, the primary species responsible for photoinduced absorption in the NIR region are expected to be the same under argon and in methanol vapor. The small spectral differences in the NIR region can therefore be attributed to the magnitude of signals observed in the presence of methanol—such large signals likely suffer from spectral distortion. As mentioned in Measurement Details, %abs values are only expected to be directly proportional to charge carrier concentration for signal sizes below ca. 10%, which is the case for the signal obtained under argon but not for the signal obtained under methanol vapor.

Overall, the spectral shape and assignment in the >1000 nm region in Figure 2 are consistent with literature reports that the absorption of photoexcited electrons increases with increasing wavelength, attributed to the free or (shallow) trapped electrons.^37,40^ Collective comparisons of the spectra obtained under argon, air, and methanol strongly suggest that the absorption of photoexcited holes increases toward shorter wavelengths in the visible region, which is again consistent with literature TAS spectra. However, the absorption peak due to photoexcited holes commonly reported in TAS was not clearly observable in the present experiment. This might be because O_2_ is not a strong electron scavenger; therefore, the populations of photoexcited holes were not enhanced to an extent that allows their absorption peak to be clearly visible, given the signal to noise ratios in the present experiment.

Furthermore, there have been some reports of (deep) trapped electrons exhibiting a broad absorption peak around 650–750 nm,^37,59^ which is also not observed presently.^1^ Bahnemann et al. noted that the absorption peak around 600–700 nm was heavily influenced by the preparation method and therefore was likely due to surface-trapped electrons.^59^ Yoshihara et al. also noted that the absorption peak assigned to trapped electrons is very sensitive to surface conditions, with nanoparticles in solution exhibiting a much stronger peak in comparison to nanocrystalline films prepared from the same particles.^37^ It may therefore be the case that the presently used TiO_2_ powder does not possess significant amounts of deep surface electron traps.

**Figure 3:**
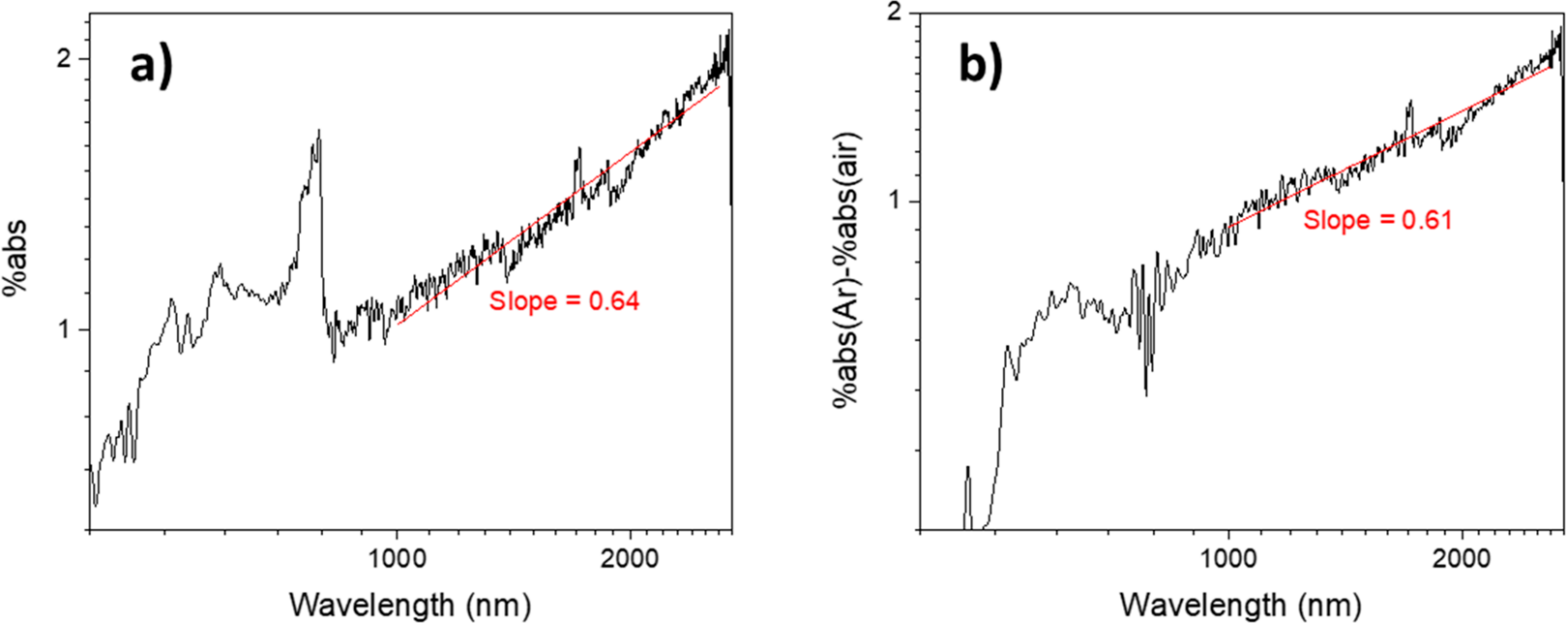
Traces of (a) argon and (b) difference (argon–air) from Figure 2a plotted on a log–log scale. The red line in each panel represents a straight-line fit through the data in the 1000–2600 nm region. The discontinuity at 800 nm is due to the detector and grating changeover at this wavelength.

Aside from absorption peaks expected for trapped charges, intraband transitions due to free carriers are expected to result in the following spectral shape:
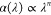
2where α(λ) is the free carrier absorption coefficient, λ is the wavelength, and *n* is a quantity related to physical characteristics of the sample, often found to take a value between 1.5 and 3.5.^62,63^ For anatase TiO_2_, Yoshihara et al. found their electron absorption spectrum to be well-fitted by *n* = 1.7, although the signal due to free electrons is superimposed with a broad peak attributed to trapped electrons,^37^ while Zhu et al. obtained a value of 1.6 for *n*.^60^ Surprisingly, Szczepankiewicz et al. also observed a λ^1.73^ dependence for the mixed-phase P25 TiO_2_ in the mid-IR region,^45^ but Yamakata et al. reported a wavenumber (ν̃) dependence of ν̃^1.5^ for Pt-loaded P25.^64^ As ν̃ ∝ 1/λ, a ν̃^1.5^ dependence is equivalent to a λ^0.67^ dependence. To identify the properties of the presently observed electrons, PIA spectra obtained under argon were replotted on a log–log scale, shown in Figure 3a (average of three repeats) and Figure S11 (individual repeats). The log–log plot appears to be approximately linear in the NIR region, but some curvature is apparent in comparison to the straight-line fit. The presence of the slight curvature suggests that these signals cannot be solely attributed to CB electrons. Nonetheless, the slopes of individual repeats that appeared approximately linear on a log–log scale were evaluated (further details in section VI in the Supporting Information). The slope was determined to be 0.63 ± 0.03, which is surprisingly close to the wavelength dependence reported by Yamakata et al. for Pt-TiO_2_ in the mid-IR region.^64^

To further compare the photoinduced absorption spectrum for TiO_2_ in argon and in air, the difference between the two spectra was also taken, shown in Figure 2a. In argon, the spectrum has contributions from all types of photoexcited charge carriers, while in air the spectrum only has contributions from charge carriers that cannot be scavenged by O_2_. As such, subtracting the spectrum in air from the spectrum in argon should yield the spectrum of one charge carrier that has been scavenged by O_2_. Although the difference spectrum in Figure 2a appears to exhibit a slight broad peak around 650 nm, the presence, position, and shape of the peak show poor reproducibility for measurements made on the same sample (Figure S6) as well as between different samples.

The difference spectra of argon and air were also plotted on a log–log scale, shown in Figure 3b (average of four repeats) and Figure S12 (individual repeats). Much better linearity is exhibited by the difference spectra in comparison to the spectra under argon, which indicates that the species responsible for the deviations from linearity are present under both argon and air, suggesting that most of the charge carriers scavenged by O_2_ are free CB electrons, which is again consistent with the finding that CB electrons are quenched by O_2_ more efficiently than trapped electrons.^60^ Through fitting data from the individual repeats (Figure S12), the slope of the difference spectra was evaluated to be 0.61 ± 0.06 (further details in section VI in the Supporting Information); thus, subtracting the spectrum obtained under air from that obtained under argon did not significantly change the slope. This indicates that the photoinduced absorption in the NIR region mainly has contributions from free CB electrons rather than species that are responsible for deviations from linearity, which is consistent with the finding that most of the photoexcited electrons observed in NIR region exist in the CB rather than as (deep) trapped states.^65^

The λ^0.61^ dependence is close to that reported by Yamakata et al.^64^ but very different from the λ^1.6–1.7^ dependence reported by other reports mentioned earlier.^37,45,60^ However, Yamakata et al.^64^ conducted their measurements in transmission mode, and the corresponding photoinduced spectra were reported in absorbance units, while Szczepankiewicz et al.^45^ used the KM transformation to analyze their reflectance data. As such, the presently obtained reflectance data were also processed using the KM transformation (Figures S13 and S14) as well as using the classical equation for absorbance (the “log(1/*r*) transformation”, Figures S15 and 16), as detailed in section VI in the Supporting Information. A log(1/*r*) transformation performed on the same data as those shown in Figure 2a resulted in slopes very similar to those found for data processed using eq 1 (%abs). However, the KM-transformed data exhibit a slope of 1.27 ± 0.06 for measurements made under argon and a slope of 1.28 ± 0.07 for the argon–air difference spectra. These values are very different from behaviors reported by all three aforementioned reports, and as mentioned in Calculation of Photoinduced Absorption, the assumptions inherent to the KM transformation are not applicable to the present system; therefore, for the remainder of this section only data processed using eq 1 will be discussed.

It may be derived that for high-frequency light in low-conductivity materials, α(λ) varies as λ^2^, while for high-frequency light in high-conductivity materials, α(λ) varies as λ^0.5^.^66^ Here, “low conductivity” means  and “high conductivity” means , where σ, ω, ε_0_, and ε_*r*_ respectively represent the material’s conductivity, the frequency of electromagnetic radiation, the vacuum permittivity, and the relative permittivity.^66^ Qualitatively, this can be interpreted as that the absorption of high-frequency light by a material possessing more dielectric character than conductor character (low conductivity) varies as λ^2^, while absorption by a material possessing more conductor character than dielectric character (high conductivity) varies as λ^0.5^. Following from this, the present results suggest that anatase TiO_2_ powder behaves more like a conductor than a dielectric under constant photoexcitation.

### Interaction between Methane and Charge Carriers in TiO_2_

**Figure 4:**
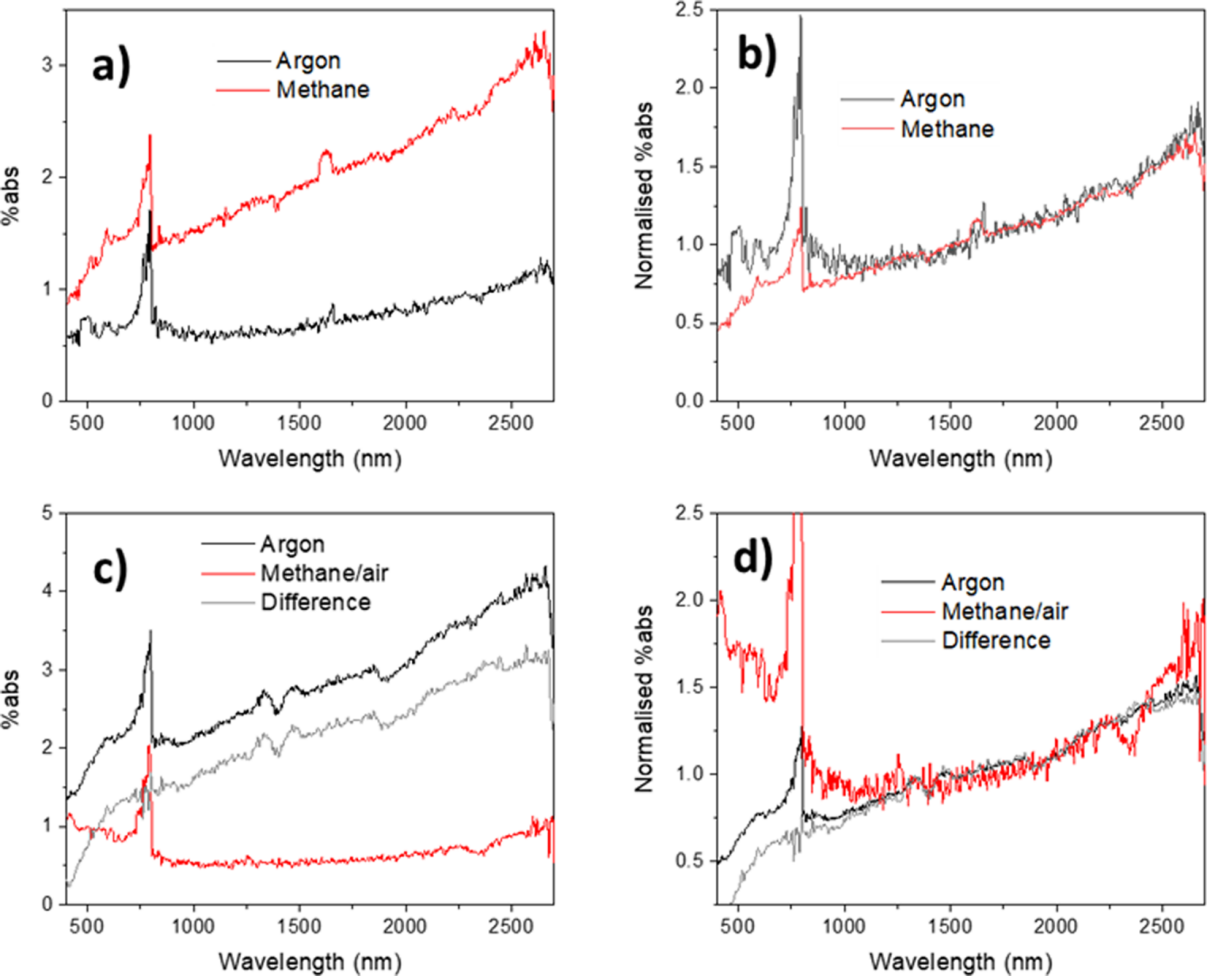
Photoinduced absorption spectra of anatase TiO_2_ powder under 365 nm excitation in the presence of (a) argon and 10% methane in argon, (c) argon and 4/1 methane/O_2_ and the corresponding normalized (using the %abs value at 1500 nm) photoinduced absorption spectra (b) for traces in (a) and (d) for traces in (c). For (c) and (d), the difference spectra (spectrum under argon minus spectrum under methane/air) are also shown in gray. The discontinuity at 800 nm is an artifact occurring at the detector and grating changeover wavelength. The average excitation intensity is estimated to be around 1 mW/cm^2^. All traces are the average of three repeats on the same sample. Data for individual repeats can be found in Figures S8 and S9 in section V in the Supporting Information for traces in (a) and (c), respectively.

Next, the reaction between photoexcited charges and methane as well as O_2_ were observed *in situ*. The photoexcited absorption characteristics of TiO_2_ in the presence of methane (with and without O_2_) will now be discussed. Figure 4a shows that, when methane is introduced, the photoinduced absorption increases across the entire measurement region, though the amount of increase is 1 order of magnitude smaller in comparison to that when methanol vapor was used. Nonetheless, Figure 4a provides strong evidence that methane is a hole scavenger; therefore, its activation over TiO_2_ likely involves oxidation directly by photoexcited holes. The small signal increase induced by methane compared to that by methanol indicates that methane is a much weaker hole acceptor in comparison to methanol, likely due to weak interactions between the TiO_2_ surface and inert, nonpolar CH_4_ molecules. This is consistent with a computational study that found the desorption energy for methanol from anatase TiO_2_ is >8 times greater than the desorption energy for methane (i.e., methanol is much more strongly adsorbed on anatase TiO_2_ than is methane).^67^ Also, other computational studies have reported that methane adsorption on anatase TiO_2_ is weak in comparison to the adsorption of other small molecules.^68,69^ Furthermore, the difficulty with dehydrogenating methane has been partially attributed to the weak interaction between methane and anatase TiO_2_,^70^ and the rate-determining step in the oxidation of methane is the dissociative adsorption of methane.^71^ Given the above, one promising route to improve the efficiency of photocatalytic methane conversion could be through the rational design of materials with enhanced affinity for methane, and strategies such as defect engineering and noble-metal doping could enhance methane adsorption.^72^ In addition, generating methanol as a preferred product in methane oxidation is rather challenging, as it can be much more easily oxidized than methane.

Upon normalizing the spectra in Figure 4a using the %abs value at 1500 nm, a good overlap between the two normalized spectra is observed in the NIR region, as shown in Figure 4b. As the previous section provided strong evidence that free electrons dominate absorption in the NIR region observed in TiO_2_ under argon, this indicates that the same populations of electrons exist in TiO_2_ in the presence of methane. On comparison of the normalized spectra in Figure 4b, it may be observed that in the visible region the difference between the two spectra increases toward shorter wavelengths. As seen in earlier discussions, it is likely the case that there are significant contributions from photoexcited holes to the absorption spectrum in the visible region; therefore, the differing shapes of the spectra acquired under argon and methane (Figure 4b) in the visible region are strong evidence that photoexcited holes are depleted in the presence of methane.

Figure 4c shows that, when methane/O_2_ (ratio 4/1, as reported previously^73^) is simultaneously introduced, the photoinduced absorption decreases in a manner similar to that observed when argon is replaced by air (Figure 2a). Similar to signal quenching by O_2_ in pure air, the magnitude of signal quenching by methane/O_2_ also decreases toward shorter wavelengths in the visible region. As seen in earlier discussions, this is indicative of a significant amount of photoexcited holes remaining in TiO_2_ under methane/O_2_. Furthermore, the difference spectrum in Figure 4c is also very similar to the difference spectrum between argon and air (Figure 2a), as shown by the comparative plot in Figure S10. This further strongly suggests that the species scavenged by 4/1 methane/O_2_ (absolute O_2_ concentration ca. 2%) are the same as the species scavenged by pure air, although there is ca. 10 times less O_2_ in the former case. The above observations collectively suggest that O_2_ preferentially adsorbs onto TiO_2_ and/or O_2_ is a much stronger electron scavenger in comparison to methane as a hole acceptor.

Normalizing the spectra in Figure 4c reveals that all three spectra roughly overlap in the NIR region, as shown in Figure 4d. The overlap between the normalized spectrum under argon and the difference spectrum (spectrum under argon minus spectrum under methane/air) is similar to the overlap between the normalized spectra under methane and argon (Figure 4b). On comparison of these normalized spectra, the difference spectrum behaves in a way similar to that of the spectrum acquired under methane when spectra acquired under argon are taken as a reference, which is consistent with the observation that photoexcited electrons are enhanced by methane but depleted by methane/air. On the other hand, photoexcited holes can be expected to be depleted by both methane and the methane/air mixture. If electrons and holes could be scavenged equally by air and methane, the normalized difference spectrum (argon minus methane/O_2_) should also show good overlap with the spectrum acquired under argon in the visible region. This is not observed; instead, the normalized difference spectrum (argon minus methane/air) is weaker than that under argon in the visible region (Figure 4d). As seen in earlier discussions, this difference in the visible region could be attributed to the inability of O_2_ to scavenge deep-trapped electrons that have strong signals in the visible region. However, given that photoexcited holes in anatase TiO_2_ exhibit strong TAS signals at around 400–500 nm,^37,38,40,59^ the present observation more likely indicates that photoexcited holes cannot be easily accessed by methane in the methane/air mixture, likely due to preferential adsorption of O_2_ onto anatase TiO_2_ (and/or more efficient electron scavenging by O_2_ than that of holes by methane). This is consistent with the observation that almost a third fewer methane molecules can adsorb onto TiO_2_ (P25) when the gas mixture is changed from methane in argon to 2/1 CH_4_/O_2_ in argon.^74^

Overall, the overlap between the normalized spectrum acquired under methane/air and the spectrum acquired under argon is comparatively poor (Figure 4d). This indicates that the majority of charge carriers that remain in TiO_2_ under methane/air are different from the primary charge carriers responsible for the photoinduced absorption in argon. Furthermore, when the normalized spectra are plotted on a log–log scale, as shown in Figure S17, there is significant curvature in the spectrum acquired under methane/air, indicating that the majority of photoexcited charges that remain in TiO_2_ are trapped charge carriers, consistent with the earlier observation that most of the charge carriers scavenged by O_2_ are free CB electrons. On the other hand, the spectrum acquired under argon is approximately linear while the difference spectrum exhibits good linearity, which is the same as observations made for data presented in Figure 2a and can be similarly rationalized.

### Efficiency of Charge Carrier Scavenging by Reactive Gases

As discussed in Calculation of Photoinduced Absorption, the %abs value calculated through eq 1 is directly proportional to the charge carrier concentration for %abs values smaller than ca. 10%. As such, taking the following ratio can yield information on the concentration change of photoexcited charge carriers when reactive gases are introduced, relative to under inert conditions
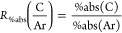
3where %abs(C) is the percentage absorption measured under a given condition (e.g., %abs(Ar) represent the %abs value measured under argon).

**Figure 5:**
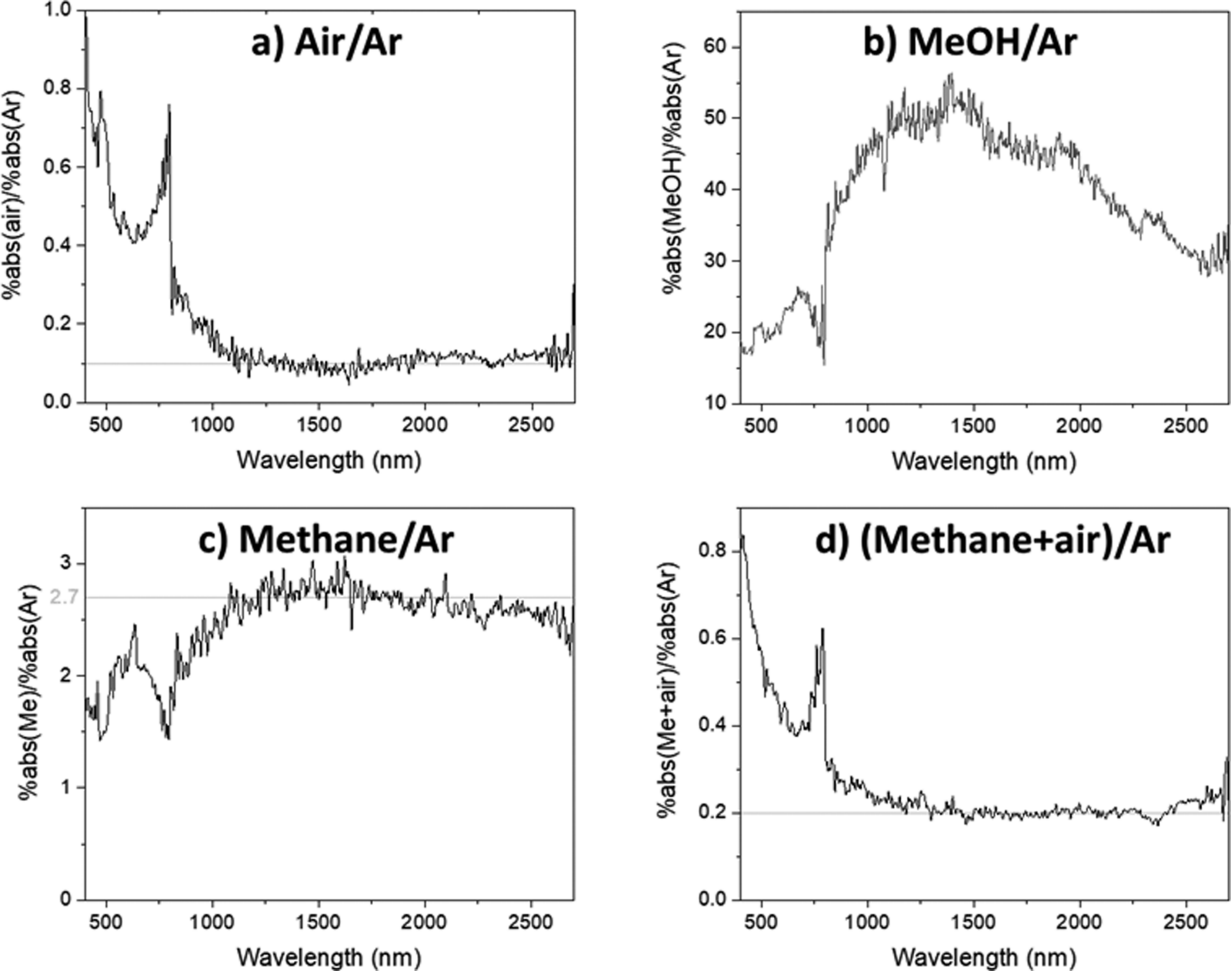
Ratio of the magnitude of photoinduced absorption over anatase TiO_2_ powder acquired under (a) dry air and argon, (b) methanol vapor (in argon) and argon, (c) methane (10% in argon) and argon, and (d) 4/1 methane/O_2_ and argon. The average intensity of the 365 nm excitation is estimated to be around 1 mW/cm^2^. The discontinuity at 800 nm is due to the detector and grating changeover at this wavelength. Apart from the trace in (b), all other traces are the average of several repeats (four repeats for (a) and (c) and three repeats for (d)) on the same sample. Data for individual repeats can be found in Figure S18 in section VII in the Supporting Information.

The linearity condition is presently satisfied for measurements made under argon, air, methane, and methane/air. Although the linearity condition is not satisfied for measurements made in the presence of methanol vapor, a similar analysis was nonetheless performed for comparison. *R*_%abs_(air/Ar), *R*_%abs_(MeOH/Ar), *R*_%abs_(methane/Ar), and *R*_%abs_((methane + air)/Ar) are plotted as a function of wavelength in parts a–d in Figure 5, respectively. It may be observed that, for wavelengths longer than ca. 1200 nm, the ratio becomes constant for all data sets apart from *R*_%abs_(MeOH/Ar), which can be attributed to spectral distortions due to extremely high %abs values measured in the presence of methanol vapor. Given the constant ratio observed for wavelengths longer than ca. 1200 nm, it is likely that all signals at wavelengths >1200 nm correspond to the same species.

To calculate how the concentration of the species responsible for signals >1200 nm changes under different environments, the average *R*_%abs_(C/Ar) value was calculated using data in the 1200–2500 nm region, as detailed in section VII in the Supporting Information. ⟨*R*_%abs_(air/Ar)⟩_1200–2500_ was calculated to be 0.10 ± 0.06 over this wide range of wavelengths, meaning that 10 ± 6% of photoexcited electrons remained in TiO_2_ after O_2_ scavenging; thus, 90 ± 6% of photoexcited electrons were scavenged by O_2_. ⟨*R*_%abs_(methane/Ar)⟩_1200–2500_ was calculated to be 2.7 ± 0.6, meaning that the population of photoexcited electrons increases by a factor of 2.7 ± 0.6 when argon is replaced by 10% methane. Assuming that the recombination probability is directly proportional to charge carrier concentrations, it may be inferred that the population of photoexcited holes decreases by a factor of 2.7 ± 0.6, and so 47.6–30.3% photoexcited holes remained in TiO_2_; thus, 52.4–69.7% (or 61 ± 9%) of photoexcited holes were scavenged by methane. Finally, ⟨*R*_%abs_((Me + air)/Ar)⟩_1200–2500_ was calculated to be 0.20 ± 0.02. Assuming that the concentration of photoexcited electrons in the absence of scavenging by O_2_ in the methane/O_2_ mixture is similar to the electron concentration under argon, it may be inferred that 80 ± 2% of photoexcited electrons were scavenged by O_2_ in the methane/air mixture. On the other hand, assuming the concentration of photoexcited electrons in the absence of scavenging by O_2_ in the methane/O_2_ mixture is similar to the electron concentration under 10% methane, and as the amount of photoexcited electrons is a factor of 2.7 ± 0.6 greater in (10%) methane compared to that in argon, ⟨*R*_%abs_((methane + air)/methane)⟩_1200–2500_ can be estimated to be 0.08 ± 0.02 (detailed calculations are outlined in section VII in the Supporting Information). It may thus be inferred that a maximum of 92 ± 2% of photoexcited electrons were scavenged by O_2_ in the methane/air mixture. Surprisingly, when the O_2_ concentration was decreased by 1 order of magnitude from 20% to 2% in a 4/1 methane/O_2_ mixture, the amount of electrons scavenged remains similar. This explains why photocatalytic oxidative coupling of methane (OCM) can effectively take place over TiO_2_-based photocatalysts even when the CH_4_/O_2_ ratio is as high as 400/1.^7^ Although the stoichiometric production of ethane and ethene from OCM respectively requires 4/1 and 2/1 ratios of methane/O_2_, providing the gases in these stoichiometric ratios does not imply that these gases are photocatalytically activated proportionally. The above results suggest that O_2_ is more easily activated than methane, and decreasing the O_2_ concentration in the gas phase by 1 order of magnitude from ca. 20% to 2% results in comparable amounts of electrons being scavenged. Together, these observations rationalize why studies with a focus on the production of value-added chemicals from methane oxidation with oxygen frequently employ a CH_4_/O_2_ ratio much higher than that required stoichiometrically.^7,22,75−78^ Methane oxidation with oxygen can result in a range of useful value-added products, including ethane, methanol, and formaldehyde.^12^ The stoichiometry of reactions between methane and oxygen ranges from 1/2 CH_4_/O_2_ (total oxidation to CO_2_) to 4/1 CH_4_/O_2_ (oxidative coupling to ethane),^12^ but many studies with a focus on production of value-added chemicals from methane oxidation with oxygen employs a higher CH_4_/O_2_ ratio than that required stoichiometrically for the formation of desired/observed products, often with over 10 times more CH_4_ than O_2_.^7,76−78^ This is also true for some non-TiO_2_-based photocatalysts, suggesting that the phenomenon of O_2_ being more easily activated than methane is not limited to TiO_2_-based materials.

On the other hand, it is noted here that there have also been a considerable number of studies that report photocatalytically oxidizing small amounts of methane with excess oxygen.^79−82^ However, the end goal of these studies was to effectively achieve the total oxidation of contaminant methane in the atmosphere to produce comparatively more benign CO_2_ molecules;^79−82^ therefore, using small amounts of methane with excess oxygen is close to the realistic conditions in this scenario. In addition, it is worth noting that for this application usually non-TiO_2_-based photocatalysts have been reported.

## Summary and Conclusion

Diffuse-reflectance measurements of anatase TiO_2_ powder under different conditions have demonstrated the capabilities of the present setup to measure the *in situ* absorption characteristics of photoexcited charge carriers in powder samples. It was found that, under constant excitation, the absorption of photoexcited holes increases toward shorter wavelengths in the visible region, and the absorption of photoexcited electrons increases with increasing wavelength, consistent with results derived from TAS measurements. However, we note that it is difficult to unambiguously distinguish between photoexcited holes and deep-trapped electrons that may exist in a limited small number of deep trap states that can absorb in the visible region. Also, photoinduced absorption in the NIR region is mainly due to CB electrons (and/or active shallow trapped electrons), and most of the charge carriers scavenged by O_2_ are these electrons. The present results also suggest that anatase TiO_2_ powders behave more like a conductor than a dielectric under constant photoexcitation, which is likely because there are significant populations of CB electrons in anatase TiO_2_ under constant photoexcitation; thus, electrons can be transferred quickly within the excited TiO_2_ semiconductor.

Applying the aforementioned system to measure anatase TiO_2_ under methane provided the first experimental evidence that the initial step of methane activation under the present condition over TiO_2_ involves oxidation by photoexcited holes. However, methane was found to be a much weaker hole acceptor in comparison to methanol, as expected from the inert and nonpolar nature of the methane molecule. This also indicates that the production of methanol through methane oxidation is rather challenging, which is the reason cocatalysts are crucial for methane oxidation to methanol by TiO_2_. Interestingly, the photoinduced absorption of TiO_2_ in 4/1 methane/O_2_ (absolute O_2_ concentration c.a. 2%) is significantly decreased relative to the absorption measured under argon, which is similar to the change observed upon changing the atmosphere from argon to air. This suggests that O_2_ preferentially adsorbs onto TiO_2_ and/or that O_2_ is a much stronger electron scavenger than methane is as a hole acceptor. Given this, one promising route to improve the efficiency of photocatalytic methane conversion could be through the rational surface engineering of materials to improve their affinity for methane.

Using the PIA data >1200 nm, it was calculated that 90 ± 6% of photoexcited electrons are scavenged by O_2_ (in dry air), 61 ± 9% of photoexcited holes are scavenged by methane (10% in argon), and about 90% of photoexcited electrons are scavenged by 4/1 methane/O_2_ (absolute O_2_ concentration ca. 2%). Thus, when the O_2_ concentration is decreased by 1 order of magnitude (from 20% to 2%) on going from pure air to 4/1 methane/O_2_, the amount of electrons scavenged remains similar, suggesting that O_2_ is much more easily activated than is methane over anatase TiO_2_, which rationalizes the much higher methane/O_2_ ratio frequently used in practice in comparison to that required stoichiometrically for photocatalytic oxidation of methane with oxygen to form value-added chemicals.

Finally, it is noted that the present results were obtained on anatase TiO_2_ powder, but analogous experiments and analysis could be performed on other benchmark photocatalysts. This can potentially reveal if the superior methane conversion activity of a photocatalyst is due to increased interactions between photoexcited charges and methane, hence aiding in the design of improved methane conversion photocatalysts. Following this, the typical polymer photocatalyst carbon nitride powders are currently being investigated using the setup demonstrated presently.

## Supporting Information Available

This material is available free of charge on the ACS Publications Web site. The Supporting Information is available free of charge at https://pubs.acs.org/doi/10.1021/acscatal.1c01998.

Further experimental details, raw reflectance spectra, data from individual repeats used to obtain averages, further data analysis, and calculation details (PDF)
