# Supplementary material for: In Situ Investigation of Charge Performance in Anatase TiO2 Powder for Methane Conversion by Vis–NIR Spectroscopy
Source: ACS Catal. 2021 Jun 20;11(13):8226–38. doi: 10.1021/acscatal.1c01998 (PMC8291573; doi:10.1021/acscatal.1c01998)
Supplement: Supplementary file 1 — cs1c01998_si_001.pdf [file cs1c01998_si_001.pdf]

Supporting Information

In-situ investigation of charge performance in anatase TiO_2 powder for methane conversion by Vis-NIR spectroscopy

Tina Jingyan Miao,[a] Chao Wang,[a] Lunqiao Xiong,[a] Xiyi Li,[a] Jijia Xie,[a,b] and Junwang Tang [a]*

[a] Department of Chemical Engineering, University College London, Torrington Place, London, WC1E 7JE, UK

[b] Present address under Author Information

*Corresponding author: junwang.tang@ucl.ac.uk

Table of Contents

I. Xe lamp excitation intensity 1

II. XRD data for TiO_2 powder 2

III. Raw reflectance data for TiO_2 measurements 3

IV. Supplementary information for $BaSO_4$ measurements 6

V. Photoinduced absorbance spectra 6

VI. Slope analysis of photoinduced absorbance spectra plotted on log-log scale 10

VII. Change in concentration of charge carriers in TiO_2 under different atmospheres 17

References 20

I. Xe lamp excitation intensity

The focal point of the Xe-lamp-lens configuration was determined to be between 10 and 14 cm, with the beam diameter varying from c.a. 0.5 cm to 0.3-0.4 cm to 1.5 cm as the distance is varied from 10

to 12 to 14 cm. The diameter of the sample holder is c.a. 0.6 cm, thus the beam diameter of the (approximately) focused Xe lamp output is roughly matched with the sample diameter.

The Xe lamp is positioned such that the total distance travelled by the Xe lamp output from the lens to the sample is c.a. 12±2 cm. Because 1) the distance between the lens and the sample can only be estimated, 2) the Xe lamp is sometimes dismantled for another experiment then re-assembled for the present experiment, and 3) the power density varies significantly with distance from the filter-lens assembly, the optical power of the filtered and focused Xe lamp was measured at 3 different distances (10, 12, and 14 cm) from the end of its optics holder. The results are summarised in Table S1. After passing through a combination of the 325-385 nm bandpass, 365 nm bandpass, and the focusing lens, the maximum power density varies from c.a. 1 to 15 mW/cm^2.

Given that 1) the Xe lamp illumination is incident on the sample at an angle, and 2) the maximum of the Xe lamp output is unlikely to be perfectly overlapped with the small internal measurement light, the actual power density at the sample is likely to be smaller than those calculated in Table S1. The final effective power density of the 365 nm excitation light is therefore estimated to be c.a. 1 mW/cm^2.

Table S1. Power (densities) of the filtered and focused Xe lamp illumination, with the distance between the power-meter sensor head and the end of the optics holder kept at c.a. 10, 12, and 14 cm (Column 1). Power of the Xe lamp output filtered by a 365 nm bandpass are shown in Column 2. Calculated power densities are shown in Column 3, with beam diameters of 0.5 cm, 0.4 cm, and 1 cm (sensor area diameter) used for a distance of 10, 12, and 14 cm, respectively. The final power density (Column 4) was calculated through multiplying values in Column 3 by 0.62, as %T of the Thorlabs UG1 filter (325-385 nm bandpass) at 365 nm was measured to be 62%.

Distance/cm	Power/mW	Power density/mW cm^{-2}	Final power density/mW cm^{-2}
10	3.7	18.8	11.7
12	3	23.9	14.8
14	1.8	2.3	1.4

II. XRD data for TiO_2 powder

A PANalytical X'Pert Pro multipurpose x-ray diffractometer was used to record the diffraction pattern of untreated and heat-treated PC50 TiO_2 powders in reflectance mode. Measurements were made using the Cu K-alpha 1 emission line (λ = 1.542 Å, 1.6 kW) in continuous scan mode (speed = 4°/min, step size = 0.01°). The as-obtained data are shown for untreated and heat-treated PC50 TiO_2

powders in Figure S1 a) and b), respectively. Both are in agreement with the reference anatase TiO_2 XRD pattern shown in Figure S1 c).

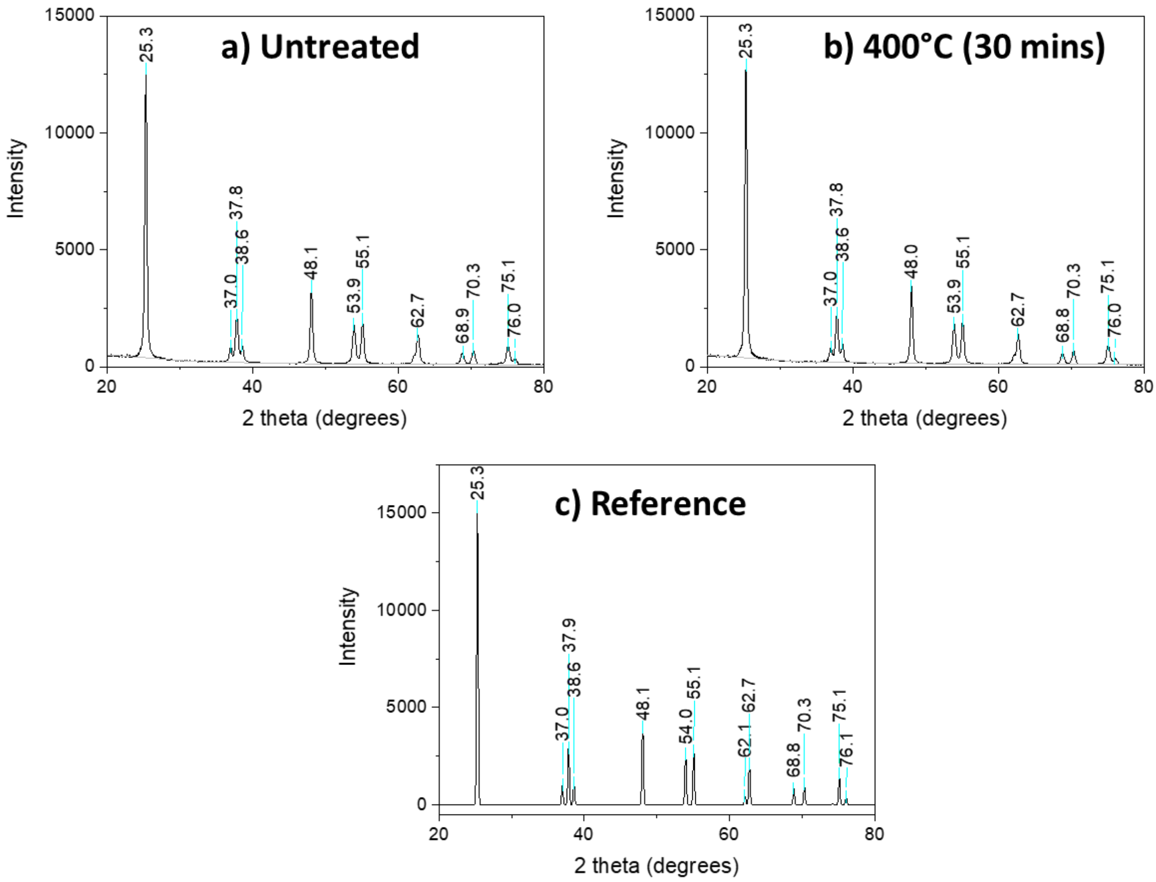

Figure S1. XRD of PC50 TiO_2 powder a) untreated and b) treated at 400°C for 30 mins. For reference, literature data[1] obtained from ICSD (Inorganic Crystal Structure Database) for anatase TiO_2 is also shown in panel c). The light grey lines in panels a) and b) are the baselines used for peak-finding.

III. Raw reflectance data for TiO_2 measurements

Raw %R spectra for anatase TiO_2 powder in the presence of air, methanol, methane, and 4/1 Methane/O_2 are respectively shown in Figure S1, Figure S2, Figure S3, and Figure S4. The slight change in overall %R going from an Argon atmosphere to air and 4/1 methane/O_2 is due to slight change in reactor height and/or orientation upon opening and closing the reactor gas valves.

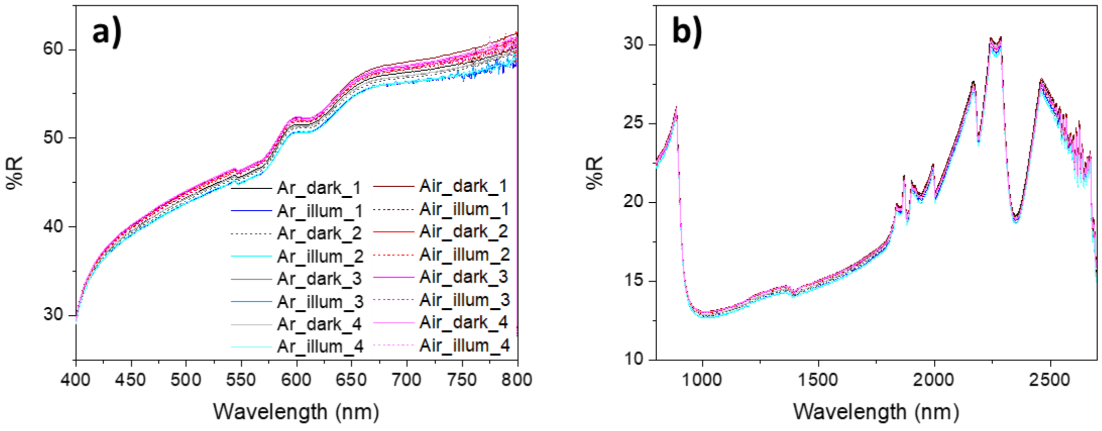

Figure S1. Raw %R data for anatase TiO_2 powder with ("illum") and without ("dark") 365 nm photoexcitation in presence of argon and (dry) air. Panels a) and b) respectively show the raw spectra in the visible region (400-800 nm) and NIR region (800-2700 nm). The legend shown in panel a) is also applicable to panel b), and the order of the legend was the order in which measurements were made (on the same sample).

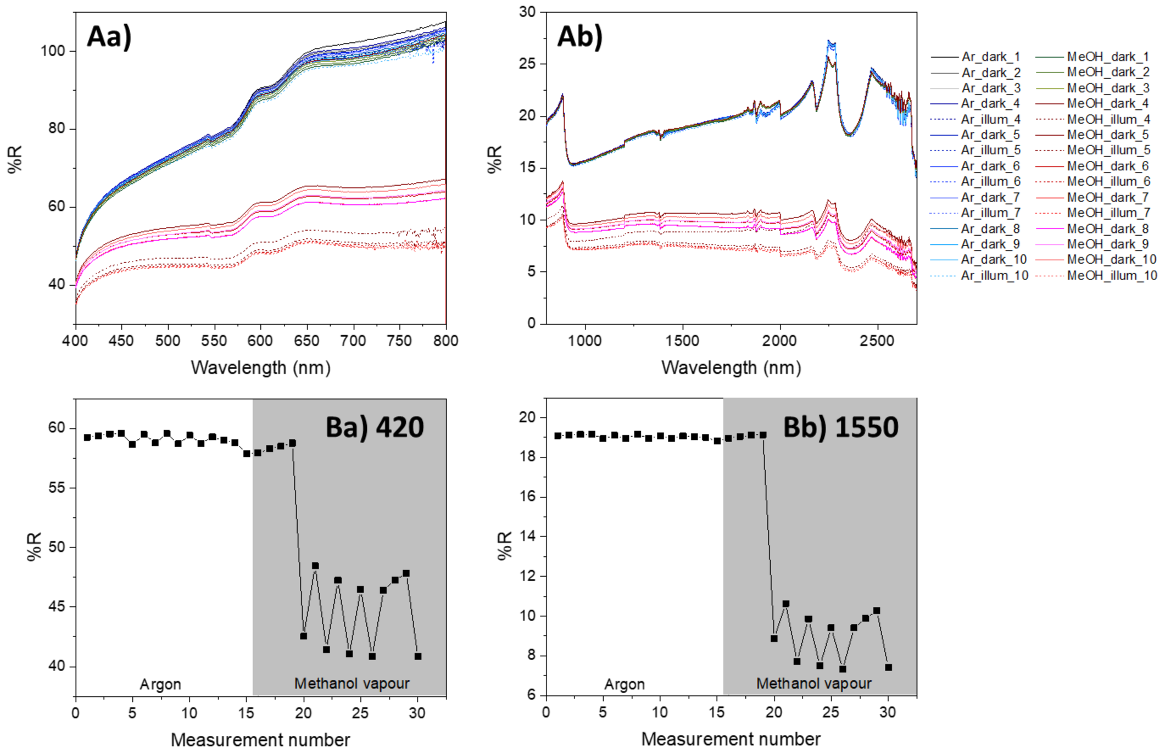

Figure S2. Raw %R data for anatase TiO_2 powder with ("illum") and without ("dark") 365 nm photoexcitation in presence of argon and methanol vapour. Panels Aa) and Ab) respectively show the raw spectra in the visible region (400-800 nm) and NIR region (800-2700 nm). Panels Ba) and Bb) respectively show evolution of the %R at 420 nm and 1550 nm as the measurements proceeded, with

the white (points 1-15) and grey (points 16-30) regions representing measurements made under argon and methanol vapour, respectively. The order of legend for panels Aa) and Ab) was the order in which measurements were made (on the same sample).

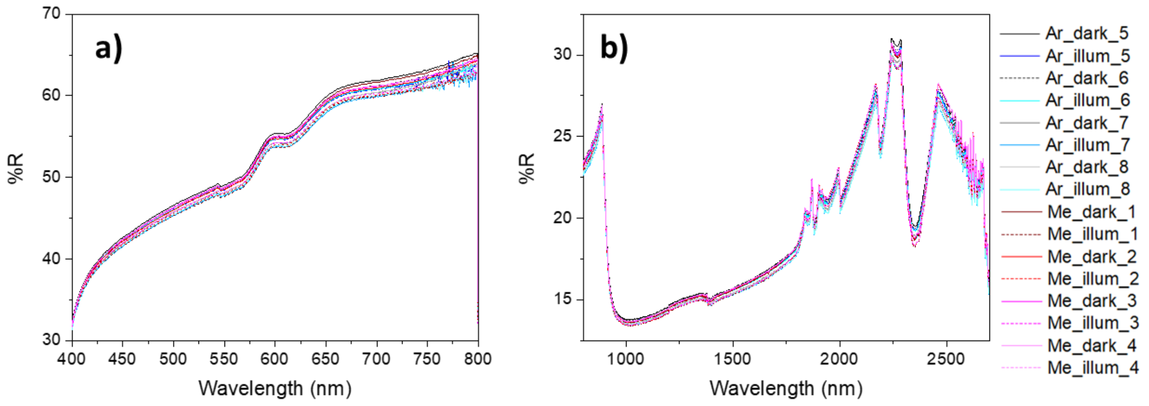

Figure S3. Raw %R data for anatase TiO_2 powder with ("illum") and without ("dark") 365 nm photoexcitation in presence of argon and 10% methane (in argon). Panels a) and b) respectively show the raw spectra in the visible region (400-800 nm) and NIR region (800-2700 nm). The order of legend shown was the order in which measurements were made (on the same sample).

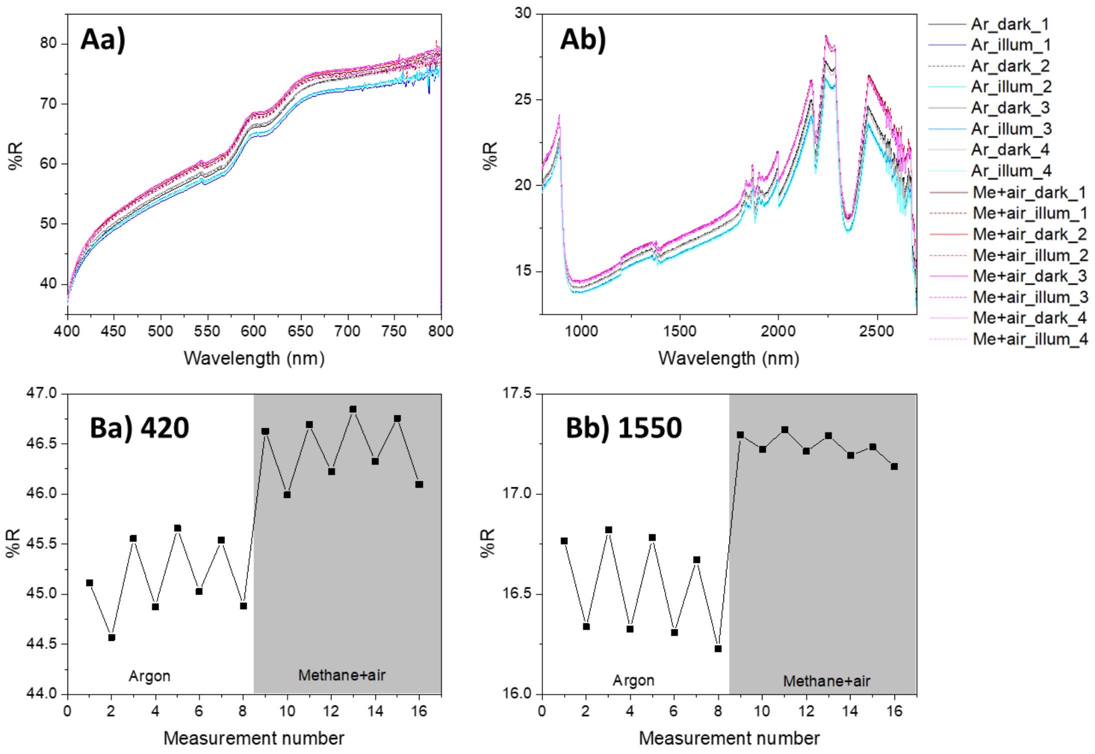

Figure S4. Raw %R data for anatase TiO_2 powder with ("illum") and without ("dark") 365 nm photoexcitation in presence of Argon and 4/1 Methane/O_2. Panels Aa) and Ab) respectively show the

raw spectra in the visible region (400-800 nm) and NIR region (800-2700 nm). Panels Ba) and Bb) respectively show evolution of the %R at 420 nm and 1550 nm as the measurements proceeded, with the white (points 1-8) and grey (points 9-16) regions representing measurements made under argon and 4/1 Methane/O_2, respectively. The order of legend for panels Aa) and Ab) was the order in which measurements were made (on the same sample).

IV. Supplementary information for $BaSO_4$ measurements

$BaSO_4$ powder was loaded into the reaction chamber sample holder and pressed flat using a spatula. Prior to measurement under each environment, the reactor chamber was purged with 150 ml/min of the relevant gas for 10 minutes, then the outlet and inlet reactor valves were closed to seal in the slightly pressurised gas. Measurements were first performed under 100% Argon, followed by (dry) air, then 10% methane in Argon.

V. Photoinduced absorbance spectra

Supplementary photoinduced absorption spectra are presented in this section. To obtain the spectra in Figure 2 a) in the main text, traces Ar_2 – Ar_4 in Figure S5 a) and traces air_2 – air_4 in Figure S5 b) were averaged over. To obtain the spectra in Figure 2 b) in the main text, all traces in Figure S6Aa) were averaged over, whilst the methanol spectrum correspond to trace MeOH_1 (the first measurement made under methanol with 365 nm excitation) in Figure S6Ab). Normalised photoinduced absorption spectra measured under 100% argon and methanol vapour (in argon) are respectively shown in Figure S6 Ba) and Bb). Individual datasets are shown as it is not appropriate to average the data acquired in the presence of methanol.

To obtain the spectra in Figure 4 a) in the main text, traces Ar_6 – Ar_8 in Figure S7 a) and traces Me_2 – Me_4 in Figure S7 b) were averaged over. To obtain the spectra in Figure 4 c) in the main text, traces Ar_2 – Ar_3 in Figure S8a) and traces Methane+air_1 – Me+air_3 in Figure S8 b) were averaged over.

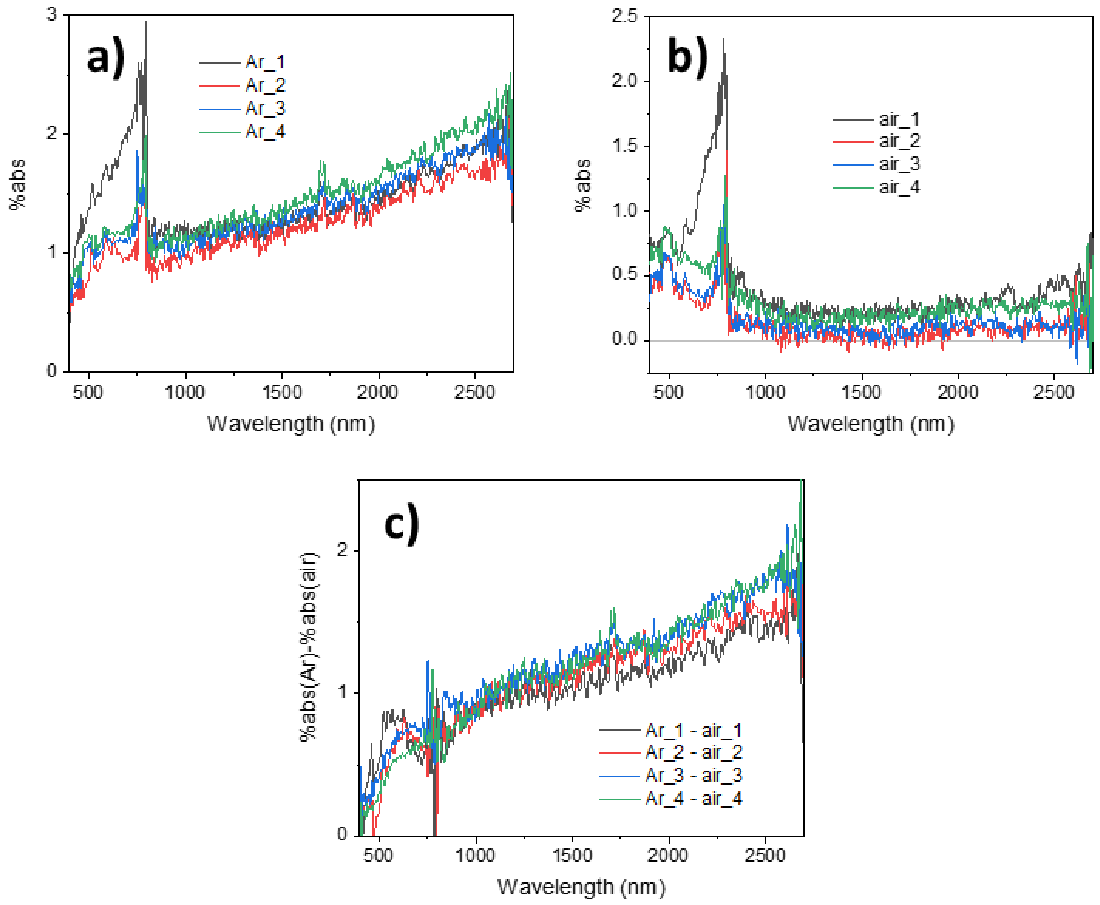

Figure S5. Photoinduced absorption spectra of anatase TiO_2 powder under 365 nm excitation in the presence of a) argon and b) dry air. The difference spectra (spectrum under argon minus spectrum under air) are shown in panel c).

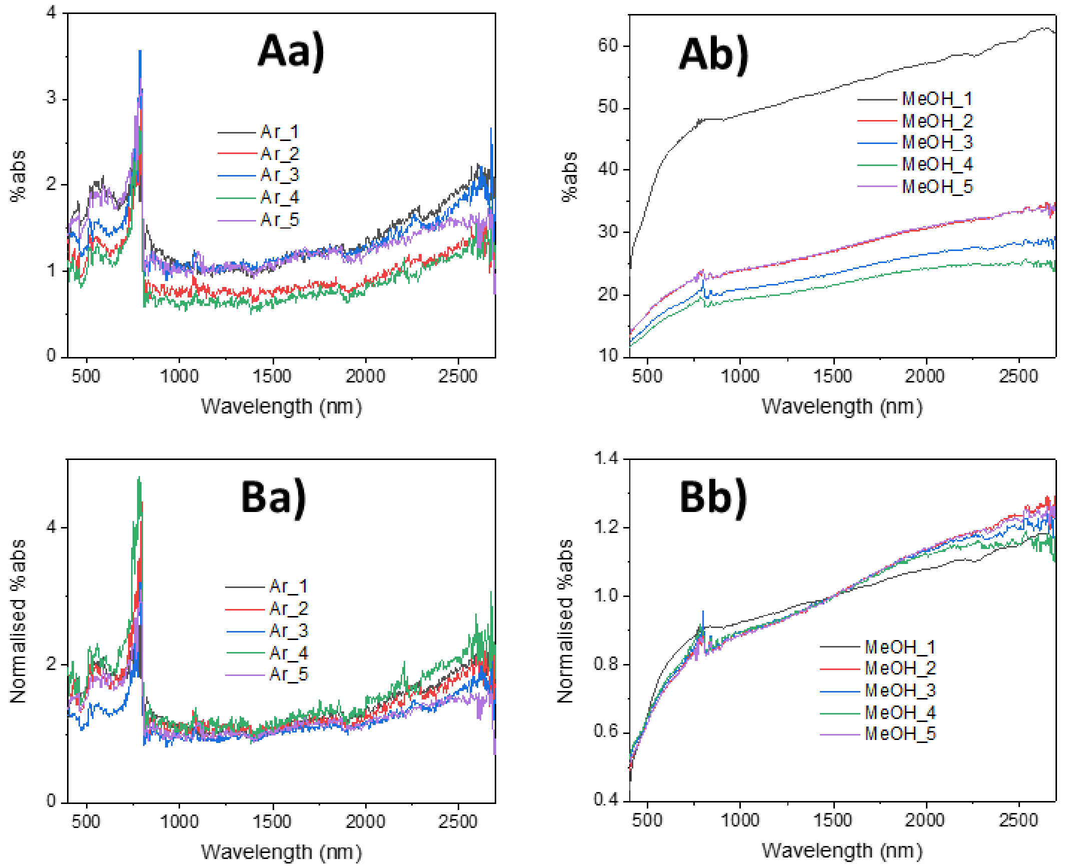

Figure S6. Photoinduced absorption spectra of anatase TiO_2 powder under 365 nm excitation in the presence of Aa) argon and Ab) methanol vapour (in argon). The normalised spectra in panels Aa) and Ab) are respectively shown in panels Ba) and Bb). The %abs values at 1500 nm were used to perform the normalisation.

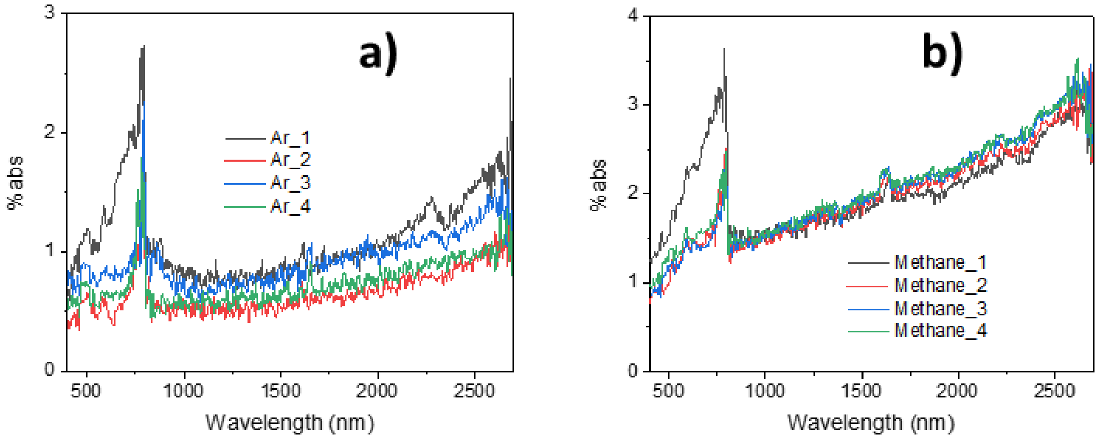

Figure S7. Photoinduced absorption spectra of anatase TiO_2 powder under 365 nm excitation in the presence of a) argon and b) 10% methane (in argon).

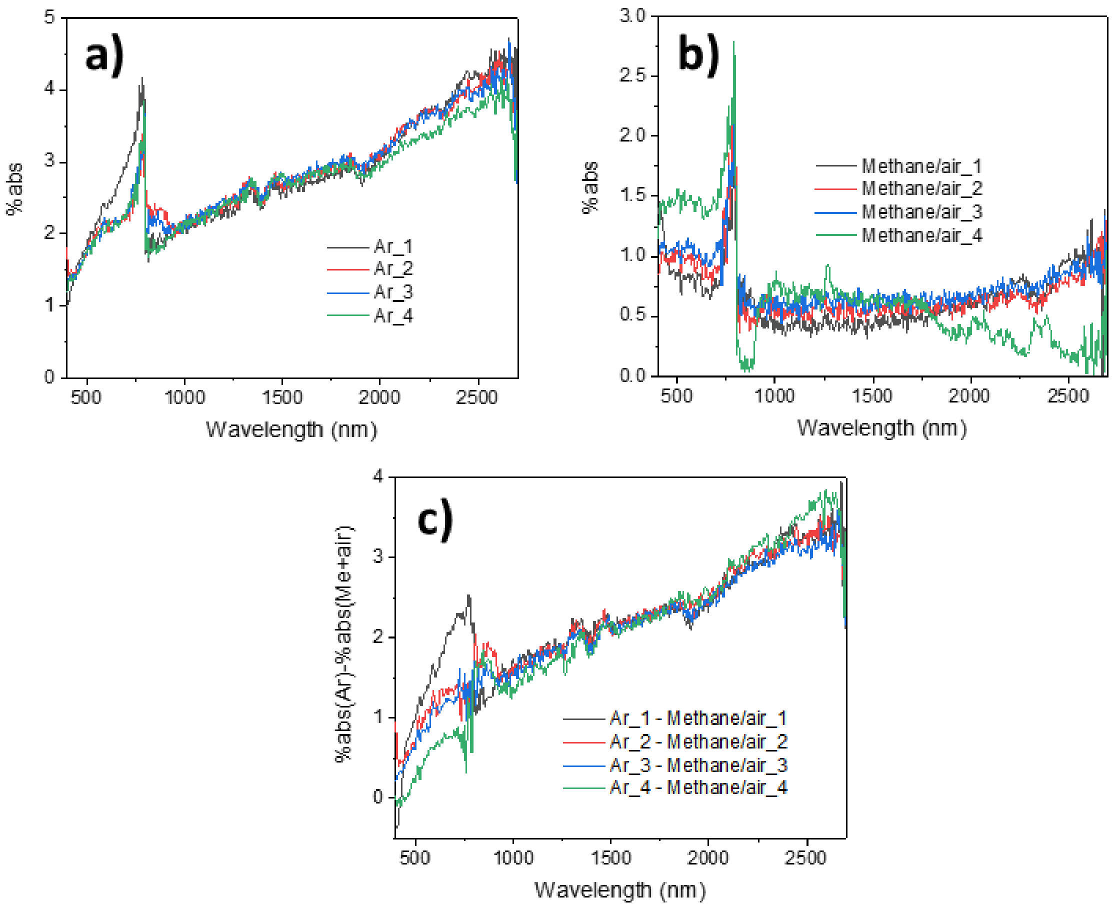

Figure S8. Photoinduced absorption spectra of anatase TiO_2 powder under 365 nm excitation in the presence of a) argon and b) 4/1 methane/O_2. The difference spectra (spectrum under argon minus spectrum under 4/1 methane/O_2) are shown in panel c).

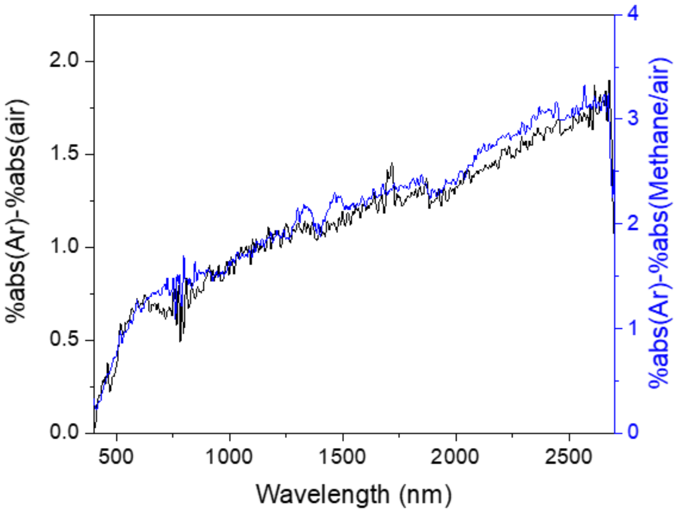

Figure S9. Comparison of the difference spectrum between argon and air in Figure 2 a) (black trace, left y-axis) and the difference spectrum between argon and 4/1 methane/O_2 in Figure 4 c) (blue trace, right y-axis).

VI. Slope analysis of photoinduced absorbance spectra plotted on log-log scale

Figure S10 shows the %abs spectra in Figure S5 Aa) re-plotted on a log-log scale. Three out of the four spectra appear to be approximately linear on a log-log scale in the NIR region, but some curvature in the plots are apparent when compared to the straight-line fit. The slopes of the approximately linear plots were evaluated. Results of linear fit through the data between 1000 and 2600 nm are shown in Table S2, and is the source of the slope value of 0.63±0.03 reported in the main text.

Figure S11 shows the %abs spectra in Figure S6 a) re-plotted on a log-log scale. Results of linear fit through the data between 1000 and 2600 nm are shown in Table S3, and is the source of the slope value of 0.61±0.06 reported in the main text.

To test the effect of expressing the photoinduced absorption amplitude in different units, the %R data was also processed using the KM and log(1/r) transformations, with the relative reflectance (r) being:

$$r = R(illum)/R(dark) \qquad \textit{(Equation S1)}$$

The KM and log(1/r) transformations were performed on the same dataset as that used to obtain the %abs spectra in Figure S5 Aa) and Ab), which in turn is the same dataset used to obtain Figure S6

a). The KM transformed spectra for the dataset obtained under Argon are shown in Figure S12, and results of linear fit through the data between 1000 and 2600 nm are shown in Table S2. The corresponding difference spectra (spectrum obtained under Argon subtracted by the spectrum obtained under air) for the KM transformed data are shown in Figure S13, and results of linear fit through the data between 1000 and 2600 nm are shown in Table S3. Finally, the log(1/r) transformed spectra for the dataset obtained under Argon and the Argon-air difference spectra are respectively shown in Figure S14 and Figure S15, with the results of linear fit through the data between 1000 and 2600 nm shown in Table S2 and Table S3, respectively.

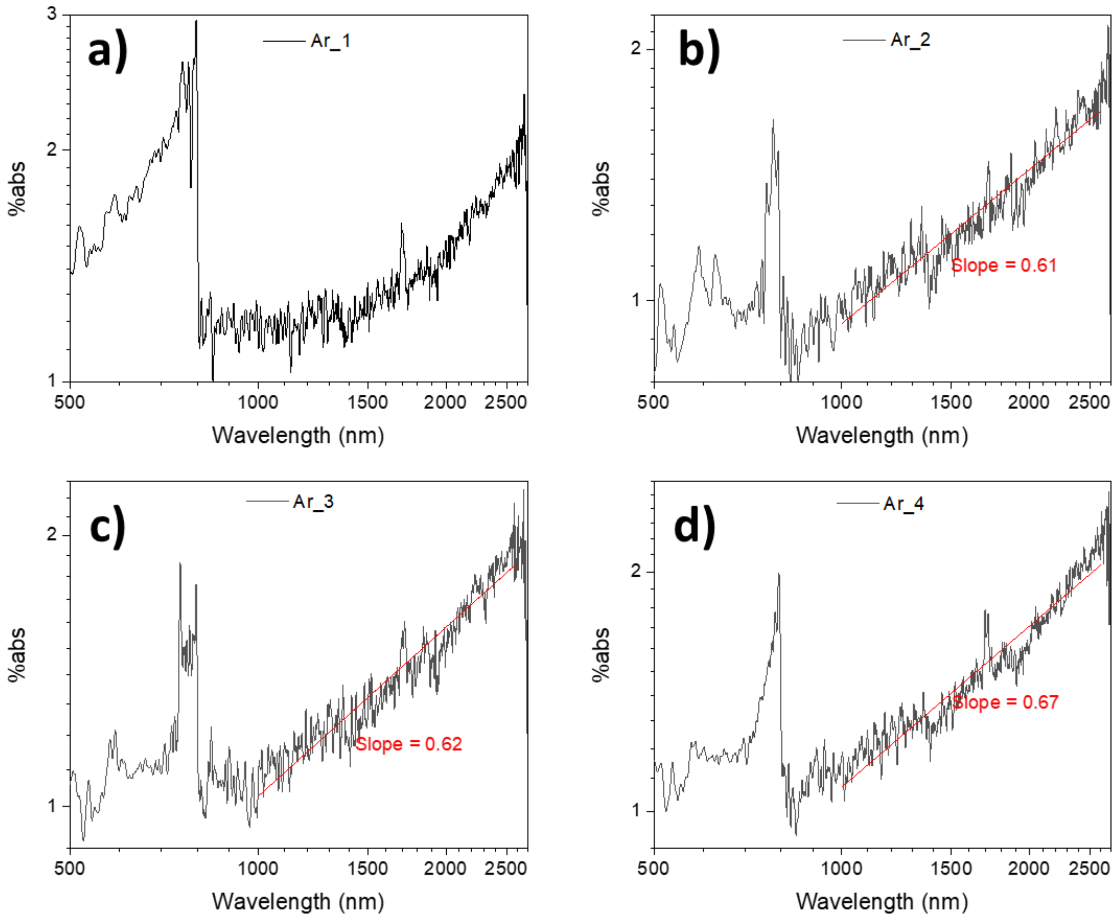

Figure S10. Trace a) Ar_1, b) Ar_2, c) Ar_3, and d) Ar_4 from Figure S5 Aa) plotted on a log-log scale. The red line in each panel represent a straight line fit through the data in the 1000-2600 nm region.

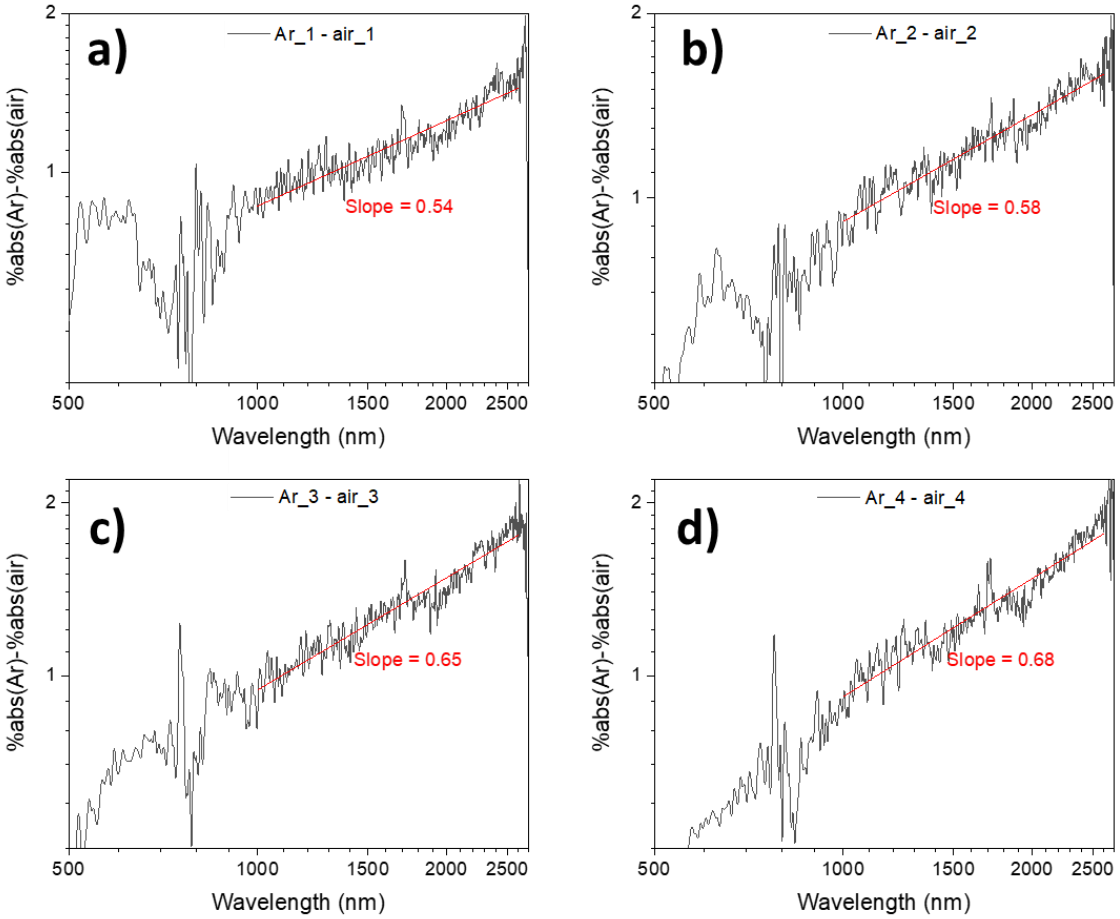

Figure S11. Trace a) Ar_1 – air_1, b) Ar_2 – air_2, c) Ar_3 – air_3, and d) Ar_4 – air_4 from Figure S6 a) plotted on a log-log scale. The red line in each panel represent a straight line fit through the data in the 1000-2600 nm region.

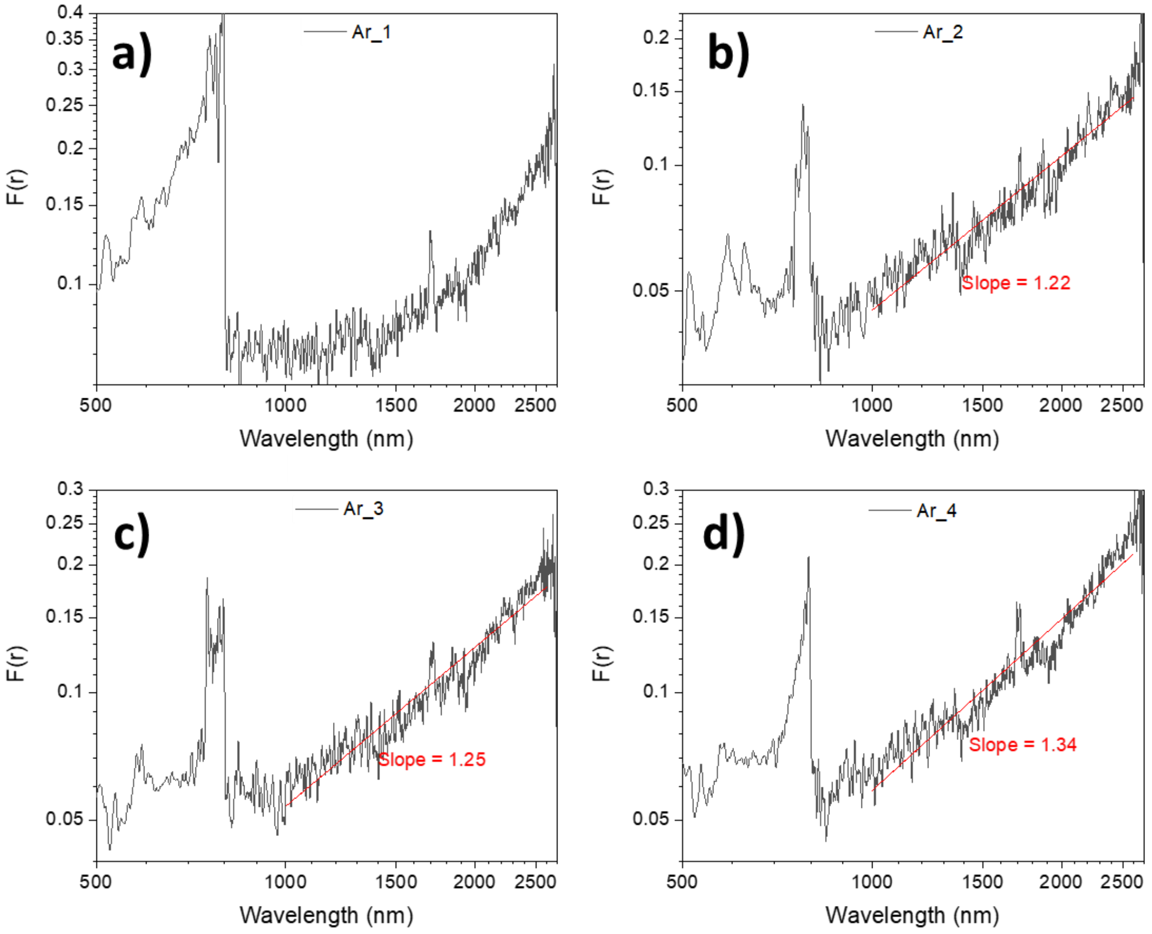

Figure S12. Photoinduced absorption spectra of anatase TiO_2 under Argon calcuated using the Kubelk-Munk transformation plotted on a log-log scale for dataset a) Ar_1, b) Ar_2, c) Ar_3, and d) Ar_4. The red line in each panel represent a straight line fit through the data in the 1000-2600 nm region.

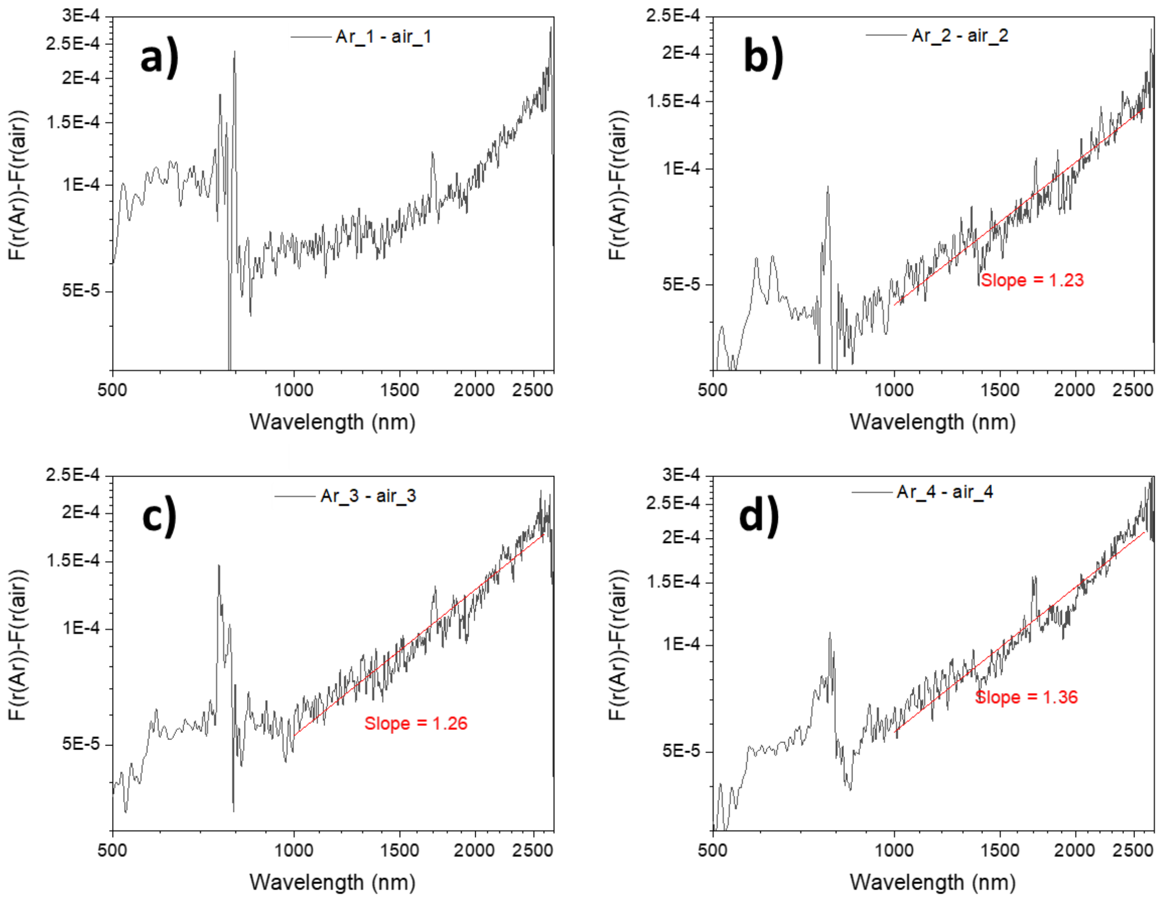

Figure S13. Difference photoinduced absorption spectra of anatase TiO_2 (spectra obtained under Argon subtracted by spectra obtained under air) calcuated using the Kubelk-Munk transformation plotted on a log-log scale for dataset a) Ar_1 – air_1, b) Ar_2 – air_2, c) Ar_3 – air_3, and d) Ar_4 – air_4. The red line in each panel represent a straight line fit through the data in the 1000-2600 nm region.

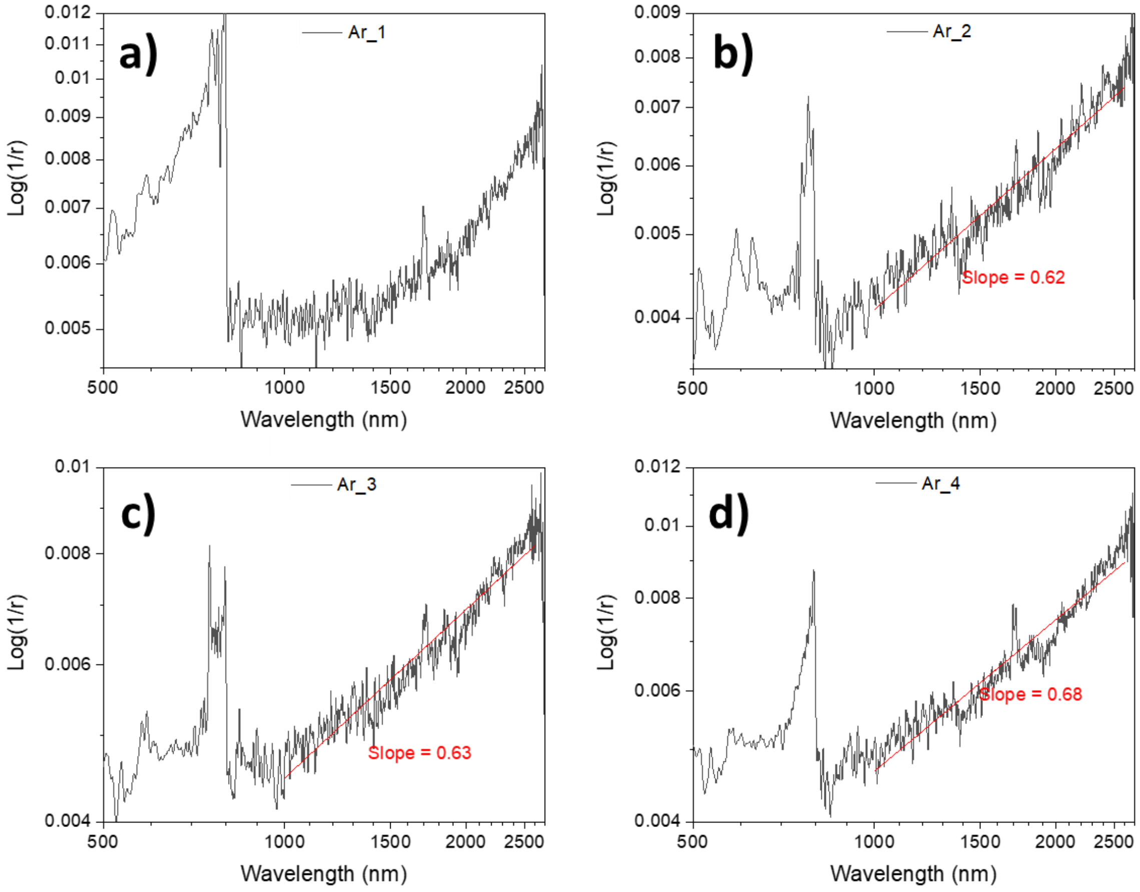

Figure S14. Photoinduced absorption spectra of anatase TiO_2 under Argon calcuated using the Log(1/r) transformation plotted on a log-log scale for dataset a) Ar_1, b) Ar_2, c) Ar_3, and d) Ar_4. The red line in each panel represent a straight line fit through the data in the 1000-2600 nm region.

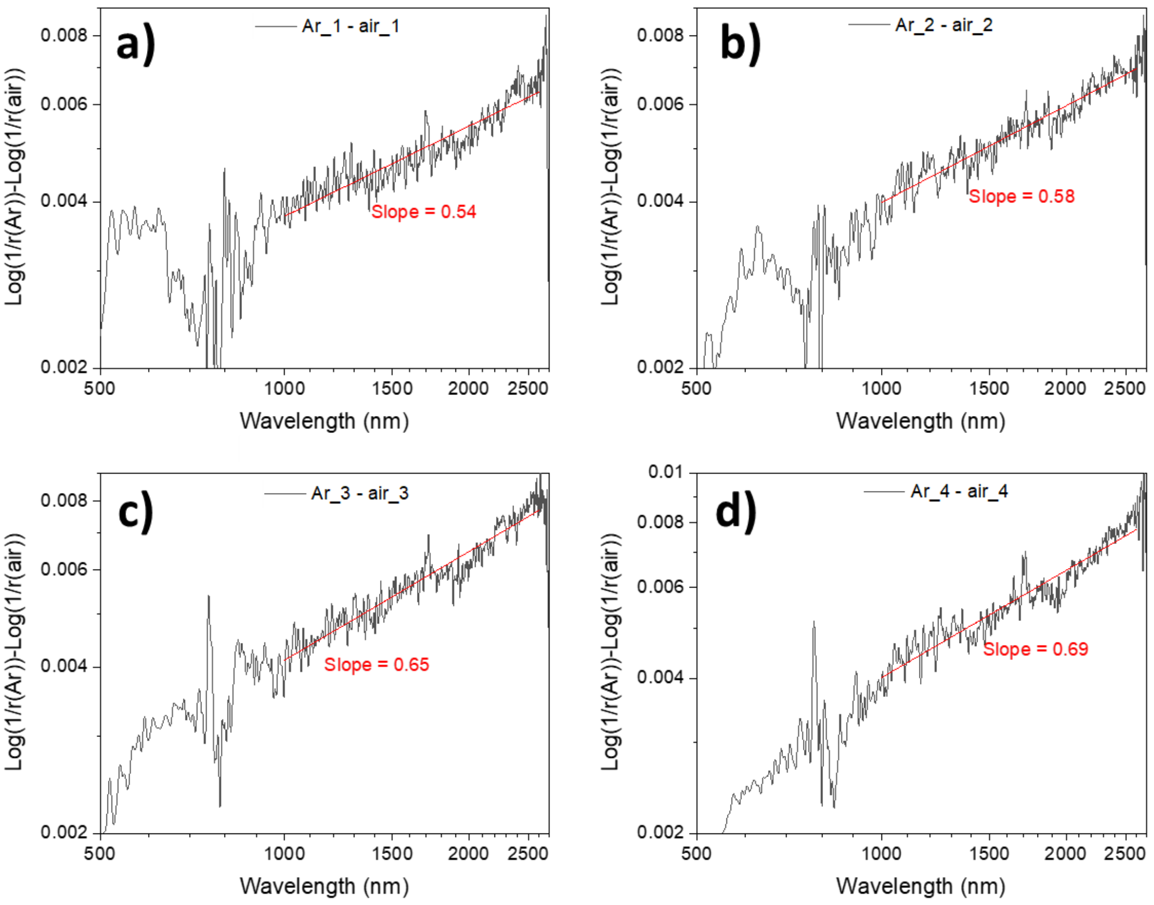

Figure S15. Difference photoinduced absorption spectra of anatase TiO_2 (spectra obtained under Argon subtracted by spectra obtained under air) calcuated using the Log(1/r) transformation plotted on a log-log scale for dataset a) Ar_1 – air_1, b) Ar_2 – air_2, c) Ar_3 – air_3, and d) Ar_4 – air_4. The red line in each panel represent a straight line fit through the data in the 1000-2600 nm region.

Table S2. Summary of slopes of photoinduced spectra obtained under Argon in the 1000-2600 nm region plotted on a log-log scale. %abs, KM, and Log(1/r) respectively represent amplitude of photoinduced signals calculated using the equation for %abs, the Kubelka-Munk transformation, and the Log(1/r) transformation. S.D. is the Standard Deviation, and n.d. means "not determined".

		**Slope/nm^{-1}**		
**Dataset**	**Range/nm**	**%abs**	**KM**	**Log(1/r)**
Ar_1	1000-2600	n.d.	n.d.	n.d.
Ar_2	1000-2600	0.61	1.22	0.62
Ar_3	1000-2600	0.62	1.25	0.63
Ar_4	1000-2600	0.67	1.34	0.68
	**Mean:**	0.63	1.27	0.64
	**S.D.:**	0.03	0.06	0.03

Table S3. Summary of slopes of difference spectra (photoinduced spectra obtained under Argon subtracted by spectra obtained under air) in the 1000-2600 nm region plotted on a log-log scale. %abs, KM, and Log(1/r) respectively represent amplitude of photoinduced signals calculated using the equation for %abs, the Kubelka-Munk transformation, and the Log(1/r) transformation. S.D. is the Standard Deviation, and n.d. means "not determined".

		Slope/nm^{-1}		
**Dataset**	**Range/nm**	**%abs**	**KM**	**Log(1/r)**
Ar_1 - air_1	1000-2600	0.54	n.d.	0.54
Ar_2 - air_2	1000-2600	0.58	1.23	0.58
Ar_3 - air_3	1000-2600	0.65	1.26	0.65
Ar_4 - air_4	1000-2600	0.68	1.36	0.69
	**Mean:**	0.61	1.28	0.62
	**S.D.:**	0.06	0.07	0.07

The spectra in Figure 4 c) in the main text were normalised (Figure 4 d)) and plotted on a log-log scale in Figure S16.

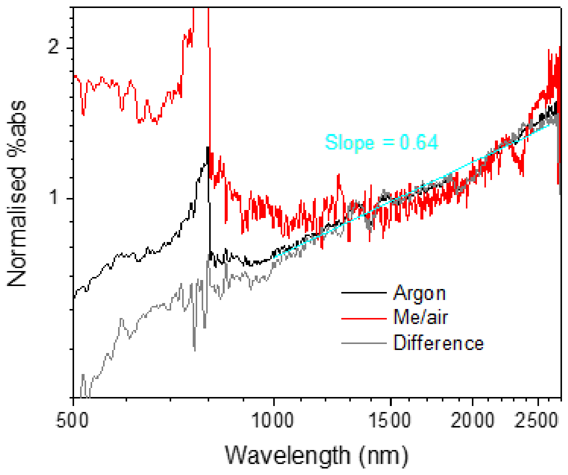

Figure S16. Data in Figure 4 d) plotted on a log-log scale. The red straight line represent a straight line fit through the Argon trace in the 1000-2600 nm region.

VII. Change in concentration of charge carriers in TiO_2 under different atmospheres

The quantities $R_{\%abs}(air/Ar)$, $R_{\%abs}(MeOH/Ar)$, $R_{\%abs}(Me/Ar)$, and $R_{\%abs}((Me + air)/Ar)$ are respectively plotted as a function of wavelength in Figure S17 a), b), c), and d) for individual repeats. All traces in Figure S17 a) and c) were averaged to obtain the average traces in Figure 5 a) and c), respectively. Figure 5 b) correspond to trace 4 in Figure S17 b). Traces 1-3 in Figure S17 d)

were averaged to obtain the average trace in Figure 5 d). Statistical analysis of the %abs ratios between 1200 and 2500 nm in Figure S17 a), c), and d) are respectively detailed in Table S4, Table S5, and Table S6. Results reported in the main text correspond to values in "Statistics on mean of datasets 1-4/1-3" at the bottom of each table.

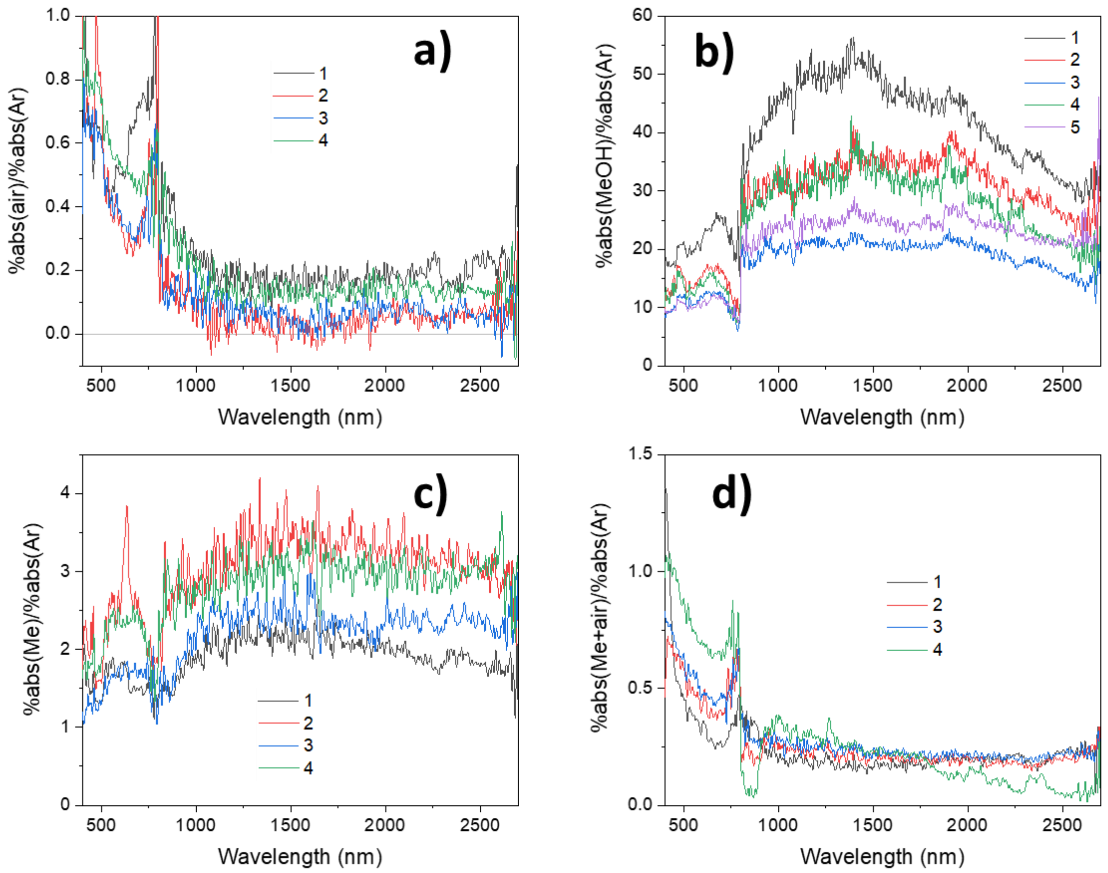

Figure S17. Photoinduced absorption ratios for photoinduced absorption spectra of anatase TiO_2 powder acquired under a) dry air and Argon, b) Methanol vapour (in Argon) and Argon, c) methane (10% in Argon) and Argon, and d) 4/1 Me/O_2 and Argon. The legend shows the repeat/measurement number. Data within each panel were obtained from the same sample, while different panels contain data collected from different samples (data from a total of 4 different samples are presently shown).

Table S4. Statistical analysis of %abs ratios shown in Figure S17 a) in the 1200-2500 nm region. The "Average" dataset is the trace obtained by averaging datasets 1-4.

**Dataset**	**Mean**	**S.D.**	**Minimum**	**Median**	**Maximum**
1	0.176	0.031	0.082	0.176	0.259
2	0.040	0.031	-0.056	0.044	0.113
3	0.064	0.029	-0.019	0.066	0.148
4	0.135	0.023	0.064	0.135	0.211
Average	0.104	0.016	0.045	0.105	0.141
**Statistics on mean of datasets 1-4:**					
**N total**	**Mean**	**S.D.**	**Minimum**	**Median**	**Maximum**
4	0.104	0.063	0.040	**0.100**	0.176

Table S5. Statistical analysis of %abs ratios shown in Figure S17 c) in the 1200-2500 nm region. The "Average" dataset is the trace obtained by averaging datasets 1-4.

**Dataset**	**Mean**	**S.D**	**Minimum**	**Median**	**Maximum**
1	2.048	0.167	1.677	2.045	2.663
2	3.312	0.235	2.764	3.280	4.208
3	2.383	0.156	1.951	2.364	2.977
4	3.017	0.190	2.390	3.010	3.660
Average	2.690	0.116	2.410	2.685	3.074
**Statistics on mean of datasets 1-4:**					
**N total**	**Mean**	**S.D**	**Minimum**	**Median**	**Maximum**
4	2.690	0.578	2.048	**2.700**	3.312

Table S6. Statistical analysis of %abs ratios shown in Figure S17 d) in the 1200-2500 nm region. The "Average" dataset is the trace obtained by averaging datasets 1-4. Dataset 4 (greyed) was excluded for calculating the "Average" dataset and for statistical analysis of the mean from the individual repeats.

**Dataset**	**Mean**	**S.D**	**Minimum**	**Median**	**Maximum**
1	0.187	0.020	0.131	0.187	0.235
2	0.198	0.021	0.147	0.196	0.276
3	0.219	0.019	0.177	0.216	0.291
4	0.170	0.074	0.045	0.154	0.376
Average	0.202	0.013	0.171	0.202	0.251
**Statistics on mean of datasets 1-3:**					
**N total**	**Mean**	**S.D**	**Minimum**	**Median**	**Maximum**
3	0.201	0.016	0.187	**0.198**	0.219

To calculate $\langle R_{\%abs}((Me + air)/Me)\rangle_{1200-2500}$, $\langle R_{\%abs}((Me + air)/Ar)\rangle_{1200-2500}$/$\langle R_{\%abs}(Me/Ar)\rangle_{1200-2500}$ were calculated, determined to be 0.091, 0.06, 0.092, and 0.056 for datasets 1, 2, 3, and 4, respectively. The mean and SD of these four values are respectively 0.075 and 0.017, which is the origin of the estimate **of 0.08±0.02** for $\langle R_{\%abs}((Me + air)/Me)\rangle_{1200-2500}$.

References

1. Ballirano, P. & Caminiti, R. Rietveld refinements on laboratory energy dispersive x-ray diffraction (EDXD) data. *J. Appl. Crystallogr.* **34**, 757–762 (2001).
